# Effects of gratitude meditation on neural network functional connectivity and brain-heart coupling

**DOI:** 10.1038/s41598-017-05520-9

**Published:** 2017-07-11

**Authors:** Sunghyon Kyeong, Joohan Kim, Dae Jin Kim, Hesun Erin Kim, Jae-Jin Kim

**Affiliations:** 1 Severance Biomedical Science Institute, Yonsei University College of Medicine, Seoul, Republic of Korea; 2 Department of Communication, Yonsei University, Seoul, Republic of Korea; 3 Brain Korea 21 PLUS Project for Medical Science, Yonsei University, Seoul, Republic of Korea; 4 Department of Psychiatry and Institute of Behavioral Science in Medicine, Yonsei University College of Medicine, Seoul, Republic of Korea

## Abstract

A sense of gratitude is a powerful and positive experience that can promote a happier life, whereas resentment is associated with life dissatisfaction. To explore the effects of gratitude and resentment on mental well-being, we acquired functional magnetic resonance imaging and heart rate (HR) data before, during, and after the gratitude and resentment interventions. Functional connectivity (FC) analysis was conducted to identify the modulatory effects of gratitude on the default mode, emotion, and reward-motivation networks. The average HR was significantly lower during the gratitude intervention than during the resentment intervention. Temporostriatal FC showed a positive correlation with HR during the gratitude intervention, but not during the resentment intervention. Temporostriatal resting-state FC was significantly decreased after the gratitude intervention compared to the resentment intervention. After the gratitude intervention, resting-state FC of the amygdala with the right dorsomedial prefrontal cortex and left dorsal anterior cingulate cortex were positively correlated with anxiety scale and depression scale, respectively. Taken together, our findings shed light on the effect of gratitude meditation on an individual’s mental well-being, and indicate that it may be a means of improving both emotion regulation and self-motivation by modulating resting-state FC in emotion and motivation-related brain regions.

## Introduction

People are subjected to a great deal of stress during daily life, and thus tend to be sensitive to negative stimuli^1^. An unhappy and stressful life is associated with decreased emotional ability and life satisfaction^2^, and also with cognitive impairments^3^. Additionally, people with high life satisfaction show greater neural connectivity among emotion-regulation-related regions during negative self-referential processing than people with low life satisfaction^4^. Therefore, it is reasonable to postulate that those who desire a happier life should be directed to reduce stress and improve mental well-being.

Positive emotion has been associated with enhanced self-regulation^5^ and resilience^6^ as well as promoting self-motivation^7^. In particular, expressing gratitude is known to promote positive mind-sets and reduce stress levels^8, 9^. Gratitude is an important component of mental healthiness throughout life, and it contributes to mental well-being^8, 10^. Gratitude has been associated with a lower risk for psychiatric disorders^11^, higher life satisfaction^10^, and wisdom^12^. More specifically, gratitude towards a parent has been associated with resilience and low levels of aggression^13^ as well as high levels of happiness and low levels of depressive symptoms^14^. Although expressing gratitude toward one’s mother is a powerful positive experience that can lead to a happier life^15^, putting this theory into practice is difficult in many cases.

Individual’s habits of resentment toward other people can be a source of life dissatisfaction^16^. Many people express more negative emotions, such as anger or resentment, than positive ones in stressful circumstances. Expression of such emotions can be mentally demanding in daily life, and associated with poorer emotional health. Furthermore, blaming others is related to a poorer mental state and emotional ill-being^17^. Therefore, developing an appropriate coping strategy to control resentment is important for managing stress and maintaining a healthy emotional life.

Although several advances have been made in understanding gratitude and resentment from a psychological point of view, few people have attempted to build a comprehensive understanding of these two emotions as agents that affect the central and autonomic nervous systems. As these systems have been studied in relation to meditation, which can temporally induce positive emotions, we referred to the biological correlates of meditation. For instance, short-term integrative body-mind training induced better physiological reactions in heart rate (HR) and skin conductance, and stronger anterior cingulate cortex (ACC) activity than simple relaxation training^18^. Long-term meditation following mindful attention training induced a longitudinal decrease in amygdala activation in response to a positive image^19^. Changes in functional connectivity (FC) within the default mode network (DMN) such as between the medial prefrontal cortex (PFC) and left inferior parietal lobule, or between the posterior cingulate cortex (PCC) and right inferior parietal lobule have been found after mindfulness meditation^20^. Furthermore, FC strength between the nucleus accumbens (NA) and dorsolateral PFC is altered after compassion training^21^.

Comparatively, no study has yet simultaneously examined neural and autonomic activities to investigate the effects of gratitude and resentment on the central and peripheral nervous systems. With respect to the effect of gratitude on brain activity, there have been two functional magnetic resonance imaging (fMRI) studies conducted. In one study, ratings of gratitude during the fMRI task were significantly correlated with ACC activity and medial PFC activity^22^. In the other study, written gratitude expressions modulated activities in the left frontoparietal, medial PFC, and occipital regions^23^. Given that altered neural activity has been reported in the PFC, ACC, amygdala, NA, and DMN regions as an effect of meditation, we postulated that brain functions embedded in these regions, such as emotional, self-referential, and reward-motivation processing, might be modulated by a psychological intervention.

In the current study, we designed two tasks–called gratitude and resentment interventions–that showed positive and negative effects on mental well-being, respectively. We then sought to identify the neurobiological consequences of these interventions, which we explored through the simultaneous acquisition of neural and autonomic activity data. We acquired fMRI data during the gratitude and resentment interventions and obtained follow-up resting-state fMRI data, in addition to the baseline resting-state fMRI scan. We obtained the autonomic data using photoplethysmography (PPG) pulse rate variability as a surrogate measurement of heartbeat^24^. We hypothesized that interventions of gratitude and resentment would activate the parasympathetic nervous system to encourage relaxation or the sympathetic nervous system to increase tension, respectively. Considering that self-referential, reward-motivation, and emotional processing are involved in these interventions, we also hypothesized that both interventions would induce modulations of neural activity, particularly through changing the default mode, emotion regulation, frontoparietal, and reward-motivation networks. Furthermore, given that these network modules are known to be interconnected, despite them being functionally segregated^25^, we tried to investigate inter-network FC using the dual-regression independent component analysis (ICA) approach.

## Results

### Data acquisition and time intervals between all consecutive fMRI scans

Neuroimaging data and behavioral scales were obtained for all participants. Unfortunately, PPG data from 3 participants was lost due to an error in the data acquisition procedure. A two-sample t-test revealed no significant differences in the time interval of the consecutive fMRI scans between the experimental set I and II (see Supplementary Table S1). Furthermore, in set I and II, paired-sample t-test revealed that the average time intervals between intervention and follow-up resting-state fMRI acquisition were 33.0 ± 10.1 and 29.8 ± 10.9 seconds for gratitude and resentment, respectively, and these intervals were not significantly different between the two interventions, regardless of experimental groups.

### HR during two intervention states

**Figure 1:**
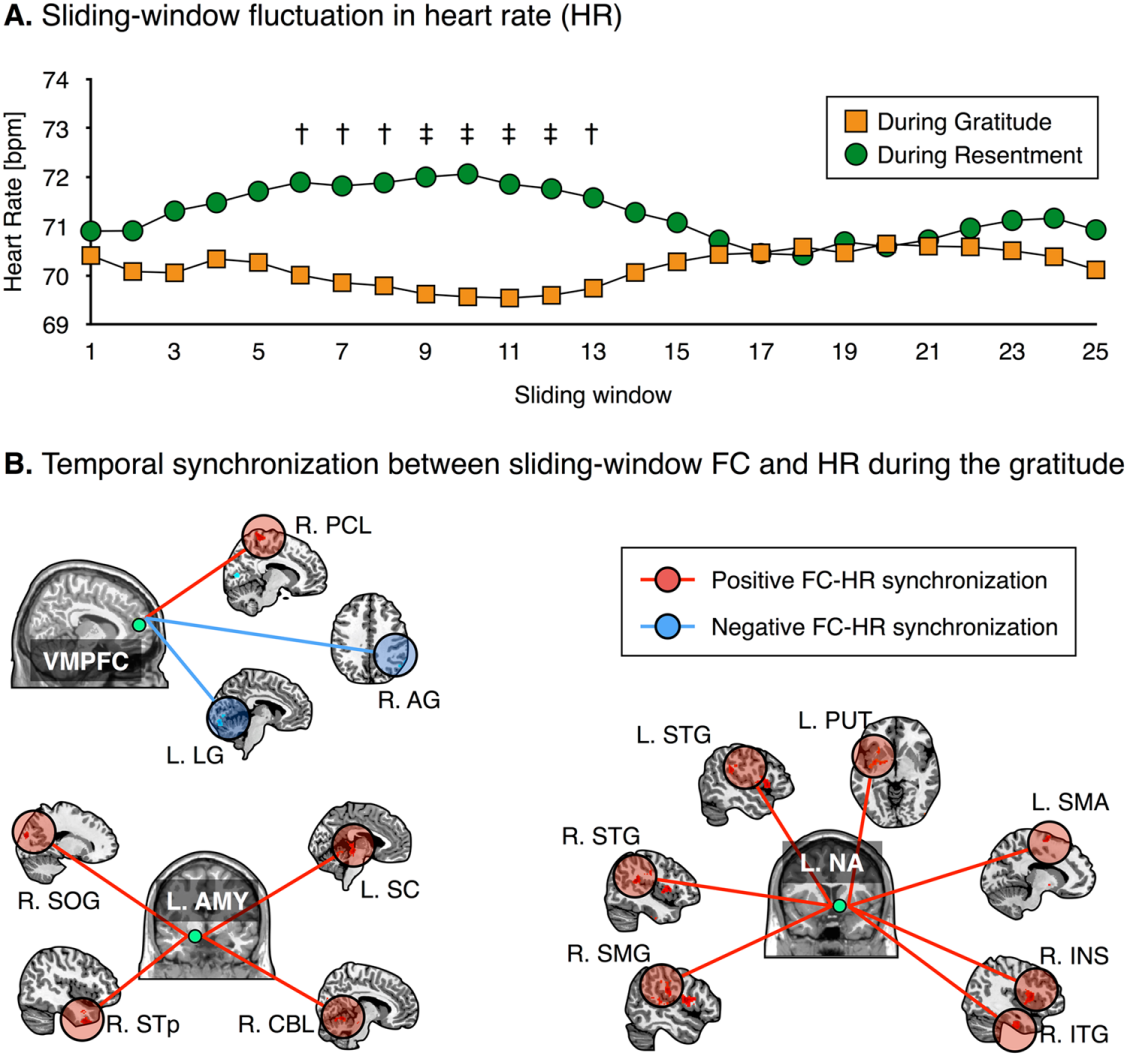
Sliding-window fluctuations in heart rate (HR) (**A**) and temporal synchronization between dynamic functional connectivity (FC) and HR during the gratitude intervention (**B**). Dagger (†) and double dagger (‡) in an inset (**A**) indicate for the significant paired sample t-test results at each sliding-window with different thresholds of *P* < 0.05 and *P* < 0.005, respectively. Peak coordinates of each cluster and statistical values are summarized in Supplementary Table S3. Abbreviations: AG, angular gyrus; bpm, beat per minutes; CBL, cerebellum; INS, insula; ITG, inferior temporal gyrus; L, left; LG, lingual gyrus; PCL, paracentral lobule; PUT, putamen; R, right; SC, superior colliculus; SMA, supplementary motor area; SMG, supramarginal gyrus; SOG, superior occipital gyrus; STG, superior temporal gyrus; STp, superior temporal pole; VMPFC, ventromedial prefrontal cortex.

Figure 1A shows the sliding-window HR values. Paired sample t-tests revealed that persistent periods of significantly decreased HR existed during the gratitude intervention than during the resentment intervention. The average HR across the sliding-windows was significantly lower during the gratitude intervention than during the resentment intervention (*t*_28_ = −2.02, *P* = 0.05), whereas the average HR was not significantly different between the two resting-states following the interventions (*t*_28_ = −0.93, *P* = 0.36).

### Temporal synchronization between FC and HR during interventions

As illustrated in Fig. 1B, we computed temporal synchronization using sliding-window FC and sliding-window fluctuation in HR during the interventions. Results show significant FC-HR synchronization values across subjects. During the gratitude intervention, the sliding-window fluctuation in HR was positively correlated with ventromedial PFC- (VMPFC)-based FC with the right paracentral lobule and negatively correlated with those with the left lingual gyrus and right angular gyrus (*P*_FWE_ < 0.05, corrected for family-wise error (FWE) rate). Moreover, positive relationships between sliding-window HR and left amygdala-based FC with the left superior colliculus, right superior occipital gyrus, right superior temporal pole, and right cerebellum were observed during the gratitude intervention (*P*_FWE_ < 0.05). Furthermore, there were positive relationships between sliding-window HR and left NA-based FC with the bilateral superior temporal gyrus, right inferior temporal gyrus, left putamen, left supplementary motor area, right supramarginal gyrus, and right insula (*P*_FWE_ < 0.05). However, no significant temporal synchronization between seed-based FC and HR during the resentment intervention was observed.

During the gratitude intervention, we observed meaningful negative coupling between sliding-window HR and inter-network FCs such as the salience–left frontoparietal network (*P*_FDR_ = 0.09). During the resentment intervention, there was significant positive coupling between sliding-window HR and inter-network FCs such as the DMN–salience network (*P*_FDR_ = 0.03).

### Functional connectivity during the interventions

**Figure 2:**
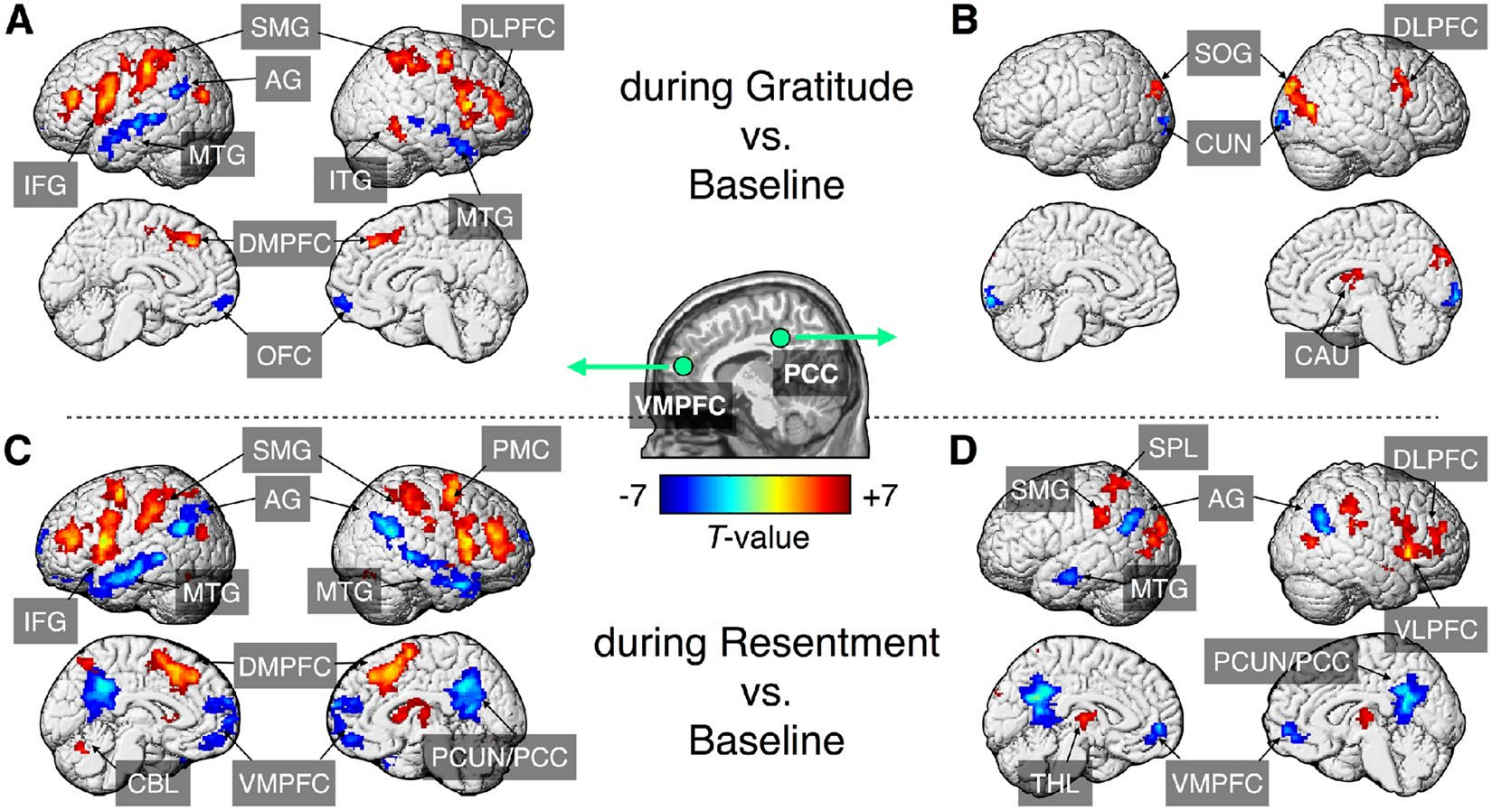
Alterations in resting-state functional connectivity during the gratitude (**A** and **B**) and resentment (**C** and **D**) interventions compared to baseline. Volume-rendered results were mapped with t-statistics for the two seed regions: (**A** and **C**) ventromedial prefrontal cortex (VMPFC) and (**B** and **D**) posterior cingulate cortex (PCC). Peak coordinates and statistical values of the clusters are summarized in Supplementary Tables S4 and S5. Abbreviation: AG, angular gyrus; CBL, cerebellum; CUN, cuneus; DLPFC, dorsolateral prefrontal cortex; DMPFC, dorsomedial prefrontal cortex; IFG, inferior frontal gyrus; ITG, inferior temporal gyrus; MTG, middle temporal gyrus; PCC, posterior cingulate cortex; PCUN, precuneus; PMC, premotor cortex; SMG, supramarginal gyrus; SOG, superior occipital gyrus; SPL, superior parietal lobule; THL, thalamus; and VLPFC, ventrolateral prefrontal cortex.

Changes in FC during the gratitude or resentment intervention relative to the baseline are presented in Supplementary Tables S4 and S5. As shown in Fig. 2, DMN connectivity, such as VMPFC-based FC with the PCC and PCC-based FC with the VMPFC, were significantly decreased by the resentment intervention (*P*_FWE_ < 0.05), but not by the gratitude intervention. During the gratitude intervention, left NA-based FC was significantly increased in the right middle temporal gyrus compared to the baseline (*P*_FWE_ < 0.05). Right NA-based FC was significantly increased in the right angular gyrus and decreased in the bilateral fusiform areas compared to the baseline (*P*_FWE_ < 0.05). During the resentment intervention, right NA-based FC was significantly increased in the precuneus (*P*_FWE_ < 0.05) and decreased in the bilateral fusiform areas compared to the baseline (*P*_FWE_ < 0.05). No significant alterations in amygdala-based FC were observed during either intervention, relative to the baseline, except significantly decreased left amygdala-based FC with the left cuneus (*P*_FWE_ < 0.05).

Table 1 compares seed-based FC between the two intervention states. During the gratitude intervention, PCC-based FC was significantly increased in the right dorsomedial PFC, left dorsolateral PFC, bilateral angular gyrus, right precuneus, and left middle temporal gyrus (*P*_FWE_ < 0.05), and VMPFC-based FC was significantly increased in the bilateral PCC and right temporoparietal junction (*P*_FWE_ < 0.05). During the resentment intervention, PCC-based FC was significantly increased in the right frontopolar PFC, right ventrolateral PFC, and right supramarginal gyrus (*P*_FWE_ < 0.05), and VMPFC-based FC was significantly increased in the right premotor cortex and left cerebellum (*P*_FWE_ < 0.05). No significant difference was observed between the two intervention states in FCs from the left amygdala, right amygdala, left NA, and right NA.

**Table 1:** Statistical comparisons of seed-based functional connectivity during gratitude and resentment interventions.

Functional connectivity	Target region	MNI coordinate, mm			Nvox	Zmax
Seed		x	y	z		
Contrast of [during Gratitude > during Resentment]						
Posterior cingulate cortex	Rt. Dorsomedial prefrontal cortex	22	34	50	1799	6.59
	Lt. Dorsolateral prefrontal cortex	−26	28	50	179	5.91
	Lt. Angular gyrus	−38	−62	36	284	6.3
	Rt. Angular gyrus	56	−64	28	701	5.46
	Rt. Precuneus	12	−52	28	2046	7.62
	Lt. Middle temporal gyrus	−62	−8	−24	136	5.99
Ventromedial prefrontal cortex	Lt. Posterior cingulate cortex	−4	−26	32	398	4.81
	Rt. Posterior cingulate cortex	2	−52	40	137	4.94
	Rt. Temporoparietal junction	54	−60	22	99	4.77
Lt. Amygdala	not significant					
Rt. Amygdala	not significant					
Lt. Nucleus accumbens	not significant					
Rt. Nucleus accumbens	not significant					
Contrast of [during Gratitude < during Resentment]						
Posterior cingulate cortex	Rt. Frontopolar prefrontal cortex	34	50	32	194	−6.15
	Rt. Ventrolateral prefrontal cortex	60	20	4	121	−5.4
	Rt. Supramarginal gyrus	64	−30	36	120	−5.6
Ventromedial prefrontal cortex	Rt. Premotor cortex	28	4	66	134	−5.62
	Lt. Cerebellum	−6	−72	−24	230	−6
Lt. Amygdala	not significant					
Rt. Amygdala	not significant					
Lt. Nucleus accumbens	not significant					
Rt. Nucleus accumbens	not significant					

Significant clusters were obtained at family-wise error rate corrected *P* < 0.05.

Abbreviation: Lt, left; MNI, Montreal Neurological Institute; Nvox, number of contiguous voxels; Rt, right; Zmax, maximum z-value within the cluster.

**Figure 3:**
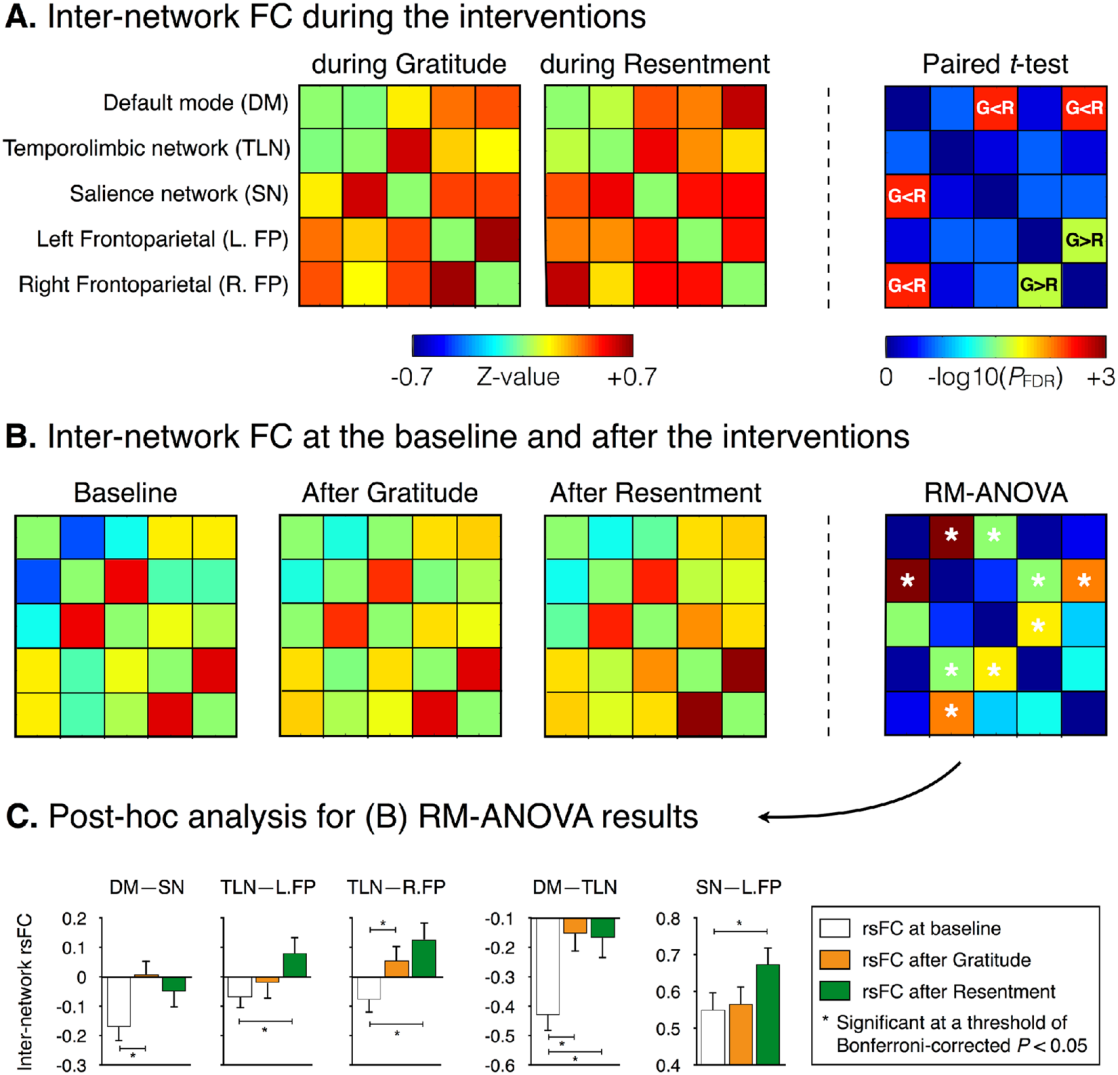
Inter-network functional connectivity (FC) among five functional networks during the gratitude and resentment interventions (**A**) and after the interventions (**B**). Average inter-network FC values across subjects before, during, and after the interventions are shown in insets (**A** and **B**). Paired sample t-tests were conducted to compare inter-network FC during the two interventions. Meanwhile, repeated-measures analysis of variance (RM-ANOVA) tests were performed to compare inter-network FC before and after the interventions. The detailed descriptions for the mean, standard deviation, and statistical value are summarized in Supplementary Tables S9 and S10. As shown in the last column in insets (**A** and **B**) statistical significances were presented in the form of −log10 (*P*_FDR_), where *P*_FDR_ is the corrected *p*-value for multiple comparisons using false discovery rate (FDR). Furthermore, post-hoc analysis was carried out for significant inter-network FCs in the RM-ANOVA test (**C**). Standard errors on a bar graph were plotted in an inset (**C**). Abbreviations: G > R (G < R) indicates that inter-network FC during the gratitude is higher (lower) than that of during the resentment; rsFC, resting-state FC.

Except for inter-network FC between the temporolimbic network and salience network as well as FC between the bilateral frontoparietal networks, all inter-network FCs were significantly increased during the both interventions, relative to the baseline (*P*_FDR_ < 0.05) (see Supplementary Table S8). Figure 3A shows results from paired-sample t-test of inter-network FCs between the two interventions. Inter-network FC between the left and right frontoparietal networks was significantly increased during the gratitude intervention (*P*_FDR_ < 0.05). In contrast, inter-network FCs of the DMN–salience network and the DMN–right frontoparietal network were significantly decreased during the gratitude intervention (*P*_FDR_ < 0.05).

### Alterations in resting-state functional connectivity after interventions

**Figure 4:**
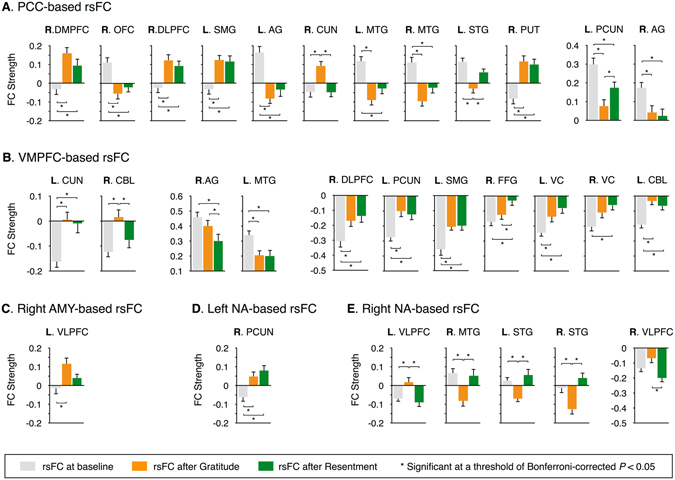
Significant seed-based resting-state functional connectivity (rsFC) and their post-hoc comparisons for (**A**) posterior cingulate cortex (PCC)-based rsFC, (**B**) ventromedial prefrontal cortex (VMPFC)-based rsFC, (**C**) right amygdala (AMY)-based rsFC, (**D**) left nucleus accumbens (NA)-based rsFC, and (**E**) right NA-based rsFC. Standard errors on bar graphs were plotted. The detailed descriptions for each cluster such as the center positions in the Montreal Neurological Institute coordinate, mean and standard deviation, cluster size, statistical value are summarized in Supplementary Table S6. Abbreviations: AG, angular gyrus; CBL, cerebellum; CUN, cuneus; DLPFC, dorsolateral prefrontal cortex; DMPFC, dorsomedial prefrontal cortex; FFG, fusiform gyrus; L, left; MTG, middle temporal gyrus; OFC, orbitofrontal gyrus; PCUN, precuneus; PUT, putamen; R, right; SMG, supramarginal gyrus; STG, superior temporal gyrus; VC, visual cortex; VLPFC, ventrolateral prefrontal cortex.

Figure 4 shows significant results from repeated-measures analysis of variance (ANOVA) and *post-hoc* analysis for the seed-based FC among resting-states at the baseline, after the gratitude intervention, and after the resentment intervention. PCC-based resting-state FC (rsFC) was significantly increased in the right dorsomedial PFC, right dorsolateral PFC, left supramarginal gyrus, and right putamen after both interventions compared to the baseline (Bonferroni-corrected *P* < 0.05). VMPFC-based rsFC was significantly increased with the left cuneus, right dorsolateral PFC, left precuneus, left supramarginal gyrus, right fusiform gyrus, left visual cortex, and left cerebellum after both interventions compared to the baseline (Bonferroni-corrected *P* < 0.05). Moreover, left NA-based rsFC with the right precuneus was significantly increased after both interventions compared to the baseline (Bonferroni-corrected *P* < 0.05). Conversely, PCC-based rsFC with the right orbitofrontal cortex, bilateral angular gyrus, right cuneus, left middle temporal gyrus, and left precuneus was significantly decreased after both interventions compared to the baseline (Bonferroni-corrected *P* < 0.05). VMPFC-based rsFC with the left middle temporal gyrus was significantly decreased after both interventions compared to the baseline (Bonferroni-corrected *P* < 0.05).

Interestingly, we observed, significant, intervention-specific increases in rsFCs between the PCC and right cuneus, between the VMPFC and right cerebellum, and between the right NA and left ventrolateral PFC after the gratitude intervention compared to both the baseline and after the resentment intervention (Bonferroni-corrected *P* < 0.05). Right amygdala-based rsFC with the left inferior frontal gyrus was significantly increased after the gratitude intervention compared to the baseline (Bonferroni-corrected *P* < 0.05). Conversely, PCC-based rsFCs with the left superior temporal gyrus and left precuneus, and right NA-based rsFCs with the right middle temporal gyrus, left superior temporal gyrus, and right superior temporal gyrus were significantly decreased after the gratitude intervention compared to both the baseline and after the resentment intervention (Bonferroni-corrected *P* < 0.05). We also found altered rsFC after the resentment intervention. VMPFC-based rsFC was significantly decreased in the right angular gyrus and the right visual cortex after the resentment intervention compared to the baseline (Bonferroni-corrected *P* < 0.05).

Figure 3B shows the result from repeated-measures ANOVA of inter-network rsFC among three conditions: the baseline, after the gratitude intervention, and after the resentment intervention, while Fig. 3C presents *post-hoc* analysis for significant inter-network rsFC. Inter-network rsFC between the DMN and temporolimbic network after gratitude and resentment interventions was significantly increased compared to that of the baseline (Bonferroni-corrected *P* < 0.05). Inter-network rsFC between the DMN and salience network was significantly increased after the gratitude intervention compared to the baseline (Bonferroni-corrected *P* < 0.05). Inter-network rsFC such as the temporolimbic–left frontoparietal, temporolimbic–right frontoparietal, and executive–left frontoparietal networks were significantly increased after the resentment intervention compared to the baseline (Bonferroni-corrected *P* < 0.05).

### Relationships between functional connectivity and behavioral variables

To explore brain regions that are significantly associated with behavioral variables, we performed linear regression analysis for the amygdala- and NA- based FC maps in the three different resting sessions: at the baseline, after the gratitude intervention, and after the resentment intervention. Table 2 shows significant relationships between bilateral amygdala-based rsFC during each resting session and subscales of the Hospital Anxiety and Depression Scale (HADS). At the baseline, the only significant result was a negative correlation between anxiety scores and right amygdala-based rsFC in the right cerebellum. After the gratitude intervention, anxiety scores were positively correlated with left amygdala-based rsFCs with the right dorsomedial PFC and right PCC, and negatively correlated with right amygdala-based rsFCs with the left dorsolateral PFC and right premotor cortex (*P*_FWE_ < 0.05). After the gratitude intervention, depression scores were positively correlated with rsFC between the bilateral amygdala and left dorsal ACC, and negatively correlated with rsFCs between the left amygdala and left inferior occipital gyrus, and between the right amygdala and bilateral fusiform gyrus (*P*_FWE_ < 0.05). After the resentment intervention, anxiety scores were negatively correlated with rsFC between the right amygdala and right dorsolateral PFC (*P*_FWE_ < 0.05). Following the resentment intervention, depression scores were negatively correlated with rsFC between the right amygdala and left fusiform gyrus, and positively correlated with rsFC between the right amygdala and left temporoparietal junction (*P*_FWE_ < 0.05).

**Table 2:** Relationships between amygdala-based functional connectivity and subscales of the hospital anxiety and depression scale.

Functional connectivity		Correlated variable	MNI coordinate, mm			Nvox	Zmax
Seed	Target region		x	y	z		
**Baseline**							
Lt. Amygdala	Not significant						
Rt. Amygdala	Rt. Cerebellum	Anxiety	22	−64	−20	354	−4.43
**After Gratitude intervention**							
Lt. Amygdala	Rt. Dorsomedial prefrontal cortex	Anxiety	10	30	58	284	4.19
	Lt. Dorsal anterior cingulate cortex	Depression	−10	42	−2	961	4.64
	Rt. Posterior cingulate cortex	Anxiety	2	−52	24	567	4.49
	Lt. Inferior occipital gyrus	Depression	−34	−104	−2	274	−3.75
Rt. Amygdala	Lt. Dorsolateral prefrontal cortex	Anxiety	−46	40	6	249	−4.48
	Lt. Dorsal anterior cingulate cortex	Depression	−10	44	−2	736	4.35
	Rt. Premotor cortex	Anxiety	42	6	60	368	−4.27
	Lt. Fusiform gyrus	Depression	−56	−72	−14	227	−4.12
	Rt. Fusiform gyrus	Depression	24	−48	−18	335	−3.77
**After Resentment intervention**							
Lt. Amygdala	Not significant						
Rt. Amygdala	Rt. Dorsolateral prefrontal cortex	Anxiety	22	48	18	359	−3.76
	Lt. Fusiform gyrus	Depression	−40	−62	−22	413	−4.22
	Lt. Tempo-parietal junction	Depression	−42	−52	46	332	3.95

Significant clusters were obtained at family-wise error rate corrected *P* < 0.05.

Abbreviation: Lt, left; MNI, Montreal Neurological Institute; Nvox, number of contiguous voxels; Rt, right; Zmax, maximum z-value within the cluster.

Table 3 shows significant linear relationships between the subscale scores of self-determination theory (SDT) and bilateral NA-based rsFC at the baseline, after the gratitude intervention, and the resentment intervention. Significant correlations between NA-based rsFC with prefrontal structures related to an individual’s reward-motivation behaviors and autonomy or relatedness scores were found after the gratitude or resentment intervention, but not at the baseline. For instance, autonomy scales were positively correlated with rsFC between the bilateral NA and bilateral dorsolateral PFC, and negatively correlated with rsFC between the left NA and left dorsal ACC after the gratitude intervention (*P*_FWE_ < 0.05). Autonomy scores were also positively correlated with rsFC between the left NA and left dorsolateral PFC after resentment intervention (*P*_FWE_ < 0.05). Relatedness scores were positively correlated with rsFC between the left NA and right dorsomedial PFC, and negatively correlated with rsFC between the bilateral NA and left VMPFC after resentment intervention (*P*_FWE_ < 0.05).

**Table 3:** Relationships between nucleus accumbens-based functional connectivity and the subscale scores of self-determination theory such as autonomy, competence, and relatedness.

Functional connectivity		Correlated variable	MNI coordinate, mm			Nvox	Zmax
Seed	Target region		x	y	z		
**Baseline**							
Lt. Nucleus accumbens	Lt. Ventromedial prefrontal cortex	Competence	2	70	6	365	−4.58
	Lt. Middle temporal pole	Autonomy	−46	0	−28	293	−3.90
	Rt. Middle temporal pole	Autonomy	34	4	−36	553	−4.43
	Rt. Middle temporal pole	Relatedness	34	8	−30	327	−4.90
	Rt. Middle temporal gyrus	Relatedness	48	−30	−8	270	3.84
	Lt. Paracentral lobule	Autonomy	−12	−22	72	282	−3.77
	Rt. Angular gyrus	Autonomy	40	−38	32	324	4.22
Rt. Nucleus accumbens	Rt. Dorsomedial prefrontal cortex	Competence	6	34	36	278	3.84
	Rt. Ventromedial prefrontal cortex	Competence	16	62	2	260	−3.34
	Lt. Middle temporal gyrus	Relatedness	−64	−24	−4	333	4.22
**After Gratitude intervention**							
Lt. Nucleus accumbens	Lt. Dorsolateral prefrontal cortex	Autonomy	−30	42	52	270	5.32
	Rt. Dorsolateral prefrontal cortex	Autonomy	42	30	48	645	4.60
	Lt. Dorsal anterior cingulate cortex	Autonomy	−8	6	30	213	−4.34
	Rt. Rolandic operculum	Autonomy	44	−26	20	224	−3.39
	Lt. Supplementary motor area	Autonomy	−14	−10	70	368	−3.74
	Rt. Supplementary motor area	Autonomy	8	−10	58	227	−4.24
	Rt. Supramarginal gyrus	Relatedness	62	−44	28	233	−3.73
	Lt. Calcarine gyrus	Autonomy	−12	−108	−2	220	4.18
	Rt. Inferior occipital gyrus	Relatedness	36	−82	−12	310	4.23
	Lt. Inferior occipital gyrus	Relatedness	−34	−82	−6	270	3.96
	Rt. Cerebellum	Competence	8	−44	−18	439	−4.07
Rt. Nucleus accumbens	Lt. Dorsolateral prefrontal cortex	Autonomy	−32	36	54	232	4.17
	Rt. Dorsolateral prefrontal cortex	Autonomy	40	26	50	378	4.40
	Lt. Paracentral lobule	Autonomy	0	−16	72	321	−3.56
	Lt. Precuneus	Relatedness	0	−66	62	221	−3.95
	Rt. Cuneus	Relatedness	12	−80	34	261	−4.40
**After Resentment intervention**							
Lt. Nucleus accumbens	Lt. Dorsolateral prefrontal cortex	Autonomy	−28	40	50	230	3.95
	Lt. Dorsolateral prefrontal cortex	Autonomy	−50	34	32	290	4.29
	Rt. Dorsomedial prefrontal cortex	Relatedness	8	36	62	228	3.79
	Lt. Ventromedial prefrontal cortex	Relatedness	−14	50	6	224	−3.84
	Lt. Posterior cingulate cortex	Autonomy	−12	−56	40	227	−3.68
Rt. Nucleus accumbens	Lt. Ventromedial prefrontal cortex	Relatedness	−18	62	10	501	−3.82
	Lt. Amygdala	Competence	−28	−2	−10	279	4.19
	Lt. Precuneus	Relatedness	−2	−58	74	274	−4.39

Significant clusters were obtained at family-wise error rate corrected *P* < 0.05.

Abbreviation: Lt, left; MNI, Montreal Neurological Institute; Nvox, number of contiguous voxels; Rt, right; Zmax, maximum z-value within the cluster.

Pearson’s correlation analyses revealed that anxiety scores were negatively correlated with inter-network rsFC between the temporolimbic and right frontoparietal networks after gratitude (*r* = −0.42, *P* = 0.018) and resentment (*r* = −0.43, *P* = 0.013) interventions, respectively. Anxiety scores were also negatively correlated with inter-network rsFC between the temporolimbic and left frontoparietal networks after resentment intervention (*r* = −0.42, *P* = 0.016), and with inter-network rsFC between the DMN and salience network after resentment intervention (*r* = −0.39, *P* = 0.029). Relatedness scores were negatively correlated with inter-network rsFC between the temporolimbic and left frontoparietal networks after resentment intervention (*r* = −0.42, *P* = 0.016), and with inter-network rsFC between the temporolimbic and salience networks after resentment intervention (*r* = −0.5, *P* = 0.003). No significant correlation was observed between inter-network rsFC at the baseline and any behavior scores.

## Discussion

To discern the effects of gratitude and resentment on the autonomic and central nervous system, we designed this study to evaluate FC, HR, and their coupling during, and after the gratitude and resentment interventions. The specific aims of our study were (i) to explore intra- and inter-network FC during and after the interventions, (ii) to identify brain-heart coupling during the interventions, (iii) to reveal the effects of the gratitude intervention on emotion- and motivation-related rsFC, and (iv) to relate rsFC to behavioral scales. Overall, our results demonstrate that patterns in default mode rsFC following the gratitude and resentment interventions were distinguishable from those in the baseline condition. Furthermore, amygdala- and NA-based rsFC, as well as inter-network rsFC among the default mode, temporolimbic, salience, and frontoparietal networks, were altered by the interventions, suggesting a modulation of neural network rsFC in emotion- and motivation-related brain networks.

During the gratitude intervention, we observed decreased HR compared to the resentment intervention. As an audio-visual guide transitions from the respiration phase (during the first minute) to the intervention phase (the next 4 minutes), the participant’s HR gradually decreased during the gratitude intervention, but increased during the resentment intervention. These features may be a result of response by the parasympathetic or sympathetic nervous systems, which inhibit and activate physiological responses, respectively^26^. Given that HR is decreased among people with high self-esteem^27^, and increased among people with high stress and anxiety^28^, our results suggest that gratitude intervention modulates heart rhythms in a way that enhances mental health. Interestingly, the persistent differences in HR during the two different interventions were observed only from the sixth to thirteenth sliding-windows. The time interval of these sequential sliding-windows was approximately 2 minutes (*i*.*e*., from 50 s to 180 s). HR was initially decreased or increased as the interventions progress, but returned to the initial state. This phenomenon may stem from perceptual desensitization, whereby increased of HR as a function of increasing emotionality returns to the initial low level^29^.

Temporal synchronization between sliding-window seed-based FC and HR was observed only during the gratitude intervention. In comparison, temporal synchronization between inter-network rsFC and HR was observed during both the gratitude and resentment interventions. Given that a decrease in HR is associated with a calm or sedative state^30^ and the amygdala is known to be a key region in emotion processing^31^, temporal synchronization of HR and amygdala-based FC with the audio-visual sensory regions such as the right superior occipital, right superior temporal pole, and left superior colliculus might be associated with emotion regulation during the gratitude intervention. Furthermore, considering that the cerebellum has been known to be associated with mental coordination, including various emotional processes^32^, temporal synchronization of HR and amygdala-based FC with the cerebellum may play a role in modulating HR during the gratitude intervention.

Our comparison of FCs from the PCC and VMPFC between during the interventions and baseline revealed minimal alteration within the DMN by the gratitude intervention, but considerable alteration by the resentment intervention. Similar results were found in the direct comparison of FCs between the gratitude and resentment interventions. During the gratitude intervention, FCs from the two functional hub regions were significantly increased in the task-negative regions, and decreased in the task-positive regions, relative to the resentment intervention. PCC-based and VMPFC-based rsFCs with task-negative regions, such as the PCC and precuneus, increased significantly during the gratitude intervention. PCC-based and VMPFC-based rsFCs with the task-positive regions, such as the supramarginal gyrus, premotor cortex, and cerebellum, decreased significantly during the resentment intervention. Performance of attention-demanding tasks routinely induces increased connectivity in certain regions of the brain and decreased connectivity in others^33^. Our rsFC-related finding may be consistent with a previous report on regional activity that neuronal deactivation within DMN regions has been found in experienced meditators, regardless of the meditation type^34^. Interestingly, PCC-dorsolateral PFC rsFC was significantly greater during the gratitude intervention than during the resentment intervention. Moreover, inter-network rsFC between the DMN and the salience network was significantly increased after the gratitude intervention compared to the baseline. DMN activity is anti-correlated with salience network activity^33^, and some studies indicate positive PCC-dorsolateral PFC rsFC during self-focused and process-oriented mental simulations^35^ and during guided mindfulness meditation practice^34^. Taken together, the modulation of intra-DMN FC during the gratitude intervention might contribute to reorganization of inter-network connectivity, such as rsFC between the DMN and the executive control network.

Although we observed no significant differences in amygdala-based FC between the gratitude and resentment interventions, we found significant relationships between emotional network rsFC after the gratitude intervention and behavioral scales. For instance, amygdala-based rsFCs with the right dorsomedial PFC and left dorsal ACC after the gratitude intervention were positively correlated with anxiety scores and depression scores, respectively. Given that individuals with low anxiety have shown significant negative amygdala–dorsomedial PFC rsFC, and that the strength of amygdala–PFC rsFC has been found to be a neural predictor of individual anxiety^36^, our gratitude intervention could play a pivotal role in reducing anxiety. Decreased dorsal ACC-amygdala rsFC has been reported in patients with emotional disorders, such as social anxiety disorder^37, 38^ and major depressive disorder^39^. Furthermore, ACC activity has been associated with social functions, such as social affect^40^ and empathy^41^. This evidence is consistent with the idea that ACC activity is facilitated by meditation^42^.

The fluctuation in rsFC between the left amygdala and right superior temporal pole was synchronized with the fluctuation in HR during the gratitude intervention. The temporal pole is engaged in object and face recognitions^43^, as well as emotional memory retrieval^44^. Meditation studies have found that mental training is accompanied by physiological modulation, such as decreased HR^18^, which, in turn, results in lower anxiety^45^ and stress^46^. Collectively, sliding-window co-fluctuations between amygdala–temporal pole rsFC and HR were observed during the gratitude intervention, and these neurophysiological coherences may play a pivotal role in reducing stress and anxiety.

After the resentment intervention, amygdala-based rsFC with the right dorsolateral PFC, and inter-network rsFC between the temporolimbic and right frontoparietal networks, were negatively correlated with anxiety scores. Increased rsFC strength in the temporolimbic–bilateral frontoparietal network was observed after the resentment intervention compared to the baseline. It is difficult to interpret the functional role of the dorsolateral PFC and frontoparietal network with regards to anxiety control because anxiety scores were negatively correlated with right amygdala–left dorsolateral PFC rsFC after the gratitude intervention, and with temporolimbic–left frontoparietal inter-network rsFC after the resentment intervention. Given that asymmetric activity in the left and right dorsolateral PFC has been found in emotion processing experiments^47, 48^, the individual’s ability to control anxiety might be lateralized to the right dorsolateral PFC. Finally, we suggest that individuals with low anxiety would display good emotional control even when experiencing a negative task, such as the resentment intervention, by modulating the amygdala–right dorsolateral PFC rsFC.

Similar to amygdala-based FC, NA-based FC did not significantly differ between the gratitude and resentment interventions. However, rsFC of this motivation network was differently altered after the two interventions. As shown in Fig. 4E, relative to rsFC after the resentment intervention, right NA-based rsFC with the bilateral ventrolateral PFC increased significantly after the gratitude intervention. Given the positive relationship between weak frontostriatal FC and poor task performance^49^, our results emphasize the importance of gratitude training in enhancing individual performance. People with high scores in relatedness showed smaller decreases in frontostriatal rsFC after the resentment intervention than those with low relatedness scores. Increases in frontostriatal rsFC have been known to be linearly associated with high-level cognition and performance^50^, and inversely related to dysfunction of inhibitory controls^51^. Therefore, our gratitude intervention might play a crucial role in improving performance on cognitive tasks. However, the strength of the NA–dorsolateral PFC rsFC after both interventions was positively correlated with autonomy scores. Considering that self-determination theory and autonomy are implicated in human motivation and behavioral self-regulation^52^, the gratitude and resentment interventions might be involved in processing motivation.

NA-based rsFCs with multiple temporal regions, such as the bilateral superior temporal gyrus and middle temporal gyrus, significantly decreased after the gratitude intervention compared to the resentment intervention. Moreover, positive synchronization of these temporostriatal rsFCs with temporal fluctuations in HR was observed during the gratitude intervention, but not during the resentment intervention. However, temporolimbic–bilateral frontoparietal inter-network rsFC after the resentment intervention was significantly increased compared to that of the baseline. Considering that the functional roles of temporal regions are associated with processing of semantic remembering^53^, NA-based rsFC after resentment intervention might be recruiting more neuronal activity to the temporolimbic regions, relative to reward processing.

Although we have discussed differences in rsFC after the gratitude and resentment interventions, there were similarities between these two conditions. For example, we observed similar patterns of rsFC modulation within the DMN regions during the two interventions relative to the baseline. In particular, the PCC-right dorsolateral PFC connection and VMPFC-bilateral supramarginal gyrus connection were found in both contexts, suggesting that there might be a shared neural mechanism between the psychological interventions. This mechanism has been considered a common element of altering participants’ emotional states by individual psychotherapy^54^. Furthermore, although NA-based FC was similar during the two interventions, it was different between resting-states after the interventions. In general, strong connections play a role in the formation of within-module connectivity, whereas weak connections play a pivotal role in the formation of between-module connectivity in the brain network modular organization^55^, and in fostering information transfer between nodes in the network^56^. Therefore, slight modulation of connections in the NA-based functional network during the two interventions might contribute to the considerable difference in rsFC after the interventions through reorganization of the functional networks from the intervention-state to the resting-state.

This study had some limitations. First, participants did not perform the audio-visual guided gratitude and resentment interventions in a calm condition due to fMRI scanning noise, which might change the degree of brain activation induced by the auditory stimuli. Second, although not very large, there was variation in the time intervals between experimental sessions. This issue was addressed in Supplementary Material S2. Third, the current study has not regressed out the possible confounding effects of the respiration. Acquisition of the respiratory data might be useful to correct physiological noise in future study. Finally, our experimental design focused on identifying only the short-term effects of the interventions.

In summary, we examined FC during, and after, the gratitude and resentment interventions, and our results indicate that modulations of neural network FC and HR occurred during, and after, both interventions. Specifically, changes in PCC-based rsFC indicate that our interventions required more neural activity in the task-positive regions than in the DMN regions. Furthermore, our findings shed light on the power of gratitude intervention on mental well-being as a means of improving not only emotion regulation, but also self-motivation, by modulating rsFC in emotion- and motivation-related brain regions. We have also provided a potential use of gratitude intervention in the treatment of patients with mood disorders or post-traumatic stress disorder. We anticipate follow-up studies will test the effects of long-term gratitude intervention training on rsFC modulation. For instance, investigation of the effect of practicing 5 minutes of gratitude meditation every day for a month on an individual’s mental health with regard to managing stress, controlling emotion, enhancing motivation, and improving life satisfaction or quality of life.

## Material and Methods

### Participants

Thirty-two healthy volunteers (mean age = 22.5 ± 2.5 years, 15 men) participated in this study. No participant had cardiac, pulmonary, metabolic, and other diseases that would cause dysfunctions in the central and autonomic nervous system. No subject had previously practiced any form of meditation. We obtained informed written consent from each subject. This study was approved by the institutional review board of Yonsei University Gangnam Severance Hospital and carried out in accordance with the Declaration of Helsinki.

### Intervention

We developed two 5-minute mental training programs called the gratitude and resentment interventions. Participants were requested to follow instructions given through an audio-visual interface within the MRI scanner. The audio-visual messages were presented in the voice of a middle-aged man as white text on a black screen. Full scripts for the interventions are provided in Supplementary Material S1. In short, the first minute of each intervention involved slow and deep breaths, focusing on respiration, to relax and calm oneself. During the gratitude intervention, participants were asked to spend the next 4 minutes focusing on a mental image of their mother. To facilitate participants to focus on the feeling of appreciation, the audio-visual messages instructed participants to tell their mothers, in their mind, how much they love and appreciate her. For the resentment intervention, participants were asked by the audio-visual messages to spend the next 4 minutes focusing on a moment or person that made them angry.

### Experimental procedure

All participants were asked to answer two kinds of self-report questionnaires before the MRI scanning procedure. We used SDT to characterize three innate psychological needs for mental well-being: competence, relatedness, and autonomy^7^. The HADS was used to evaluate participants’ state of anxiety and depression^57^.

**Figure 5:**
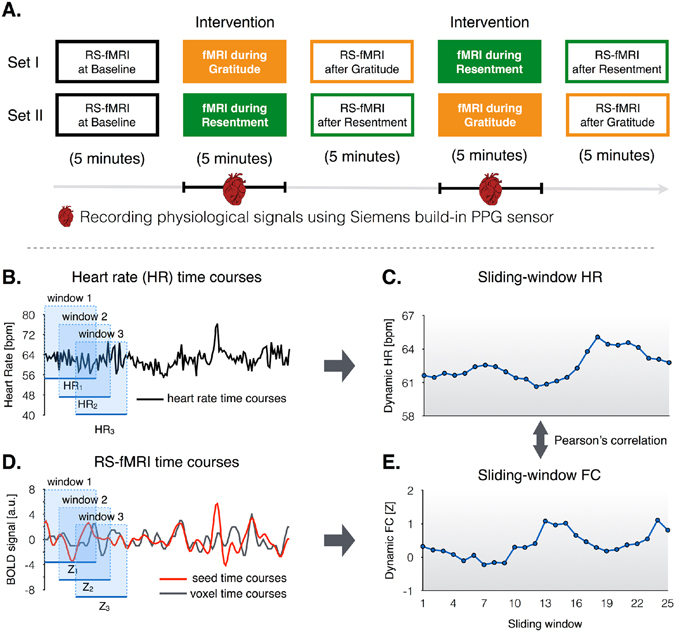
Experimental procedure (**A**). The order of experiments (set I and II) were counter balanced across participants. Illustration for how to evaluate the temporal synchronization between dynamic functional connectivity and heart rate (HR) during the interventions (**B**–**E**). Raw time courses and illustration of the sliding-windows for HR (**B**) and fMRI time series (**D**). Strategies to compute the temporal correlation between dynamic HR (**C**) and dynamic FC (**E**). Abbreviations: RS-fMRI, resting-state functional MRI; PPG, photoplethysmography.

Figure 5A shows the experimental procedures. Participants were seated comfortably for at least 5 minutes before the experiment and then underwent five sessions of fMRI experiments in the following order: baseline resting-state, the first intervention, resting-state after the first intervention, the second intervention, and resting-state after the second intervention. The order of the two interventions assigned to the experimental groups was random; either the gratitude intervention was followed by the resentment intervention (N = 17 with 8 men, assigned to set I) or the reverse order (N = 15 with 7 men, assigned to set II). We tried to minimize the time intervals between all successive sessions. In the resting-state, participants were instructed to open their eyes and watch the crosshair on the screen.

### Imaging parameters and pre-processing

All examinations were performed on a 3.0 Tesla MR scanner (Magnetom Verio, Siemens Medical Solutions). For each participant, we acquired 155 whole-brain scans using gradient-recall echo-planar imaging with the following parameters: matrix size = 64 × 64, number of slices = 30, slice order = bottom-up and interleaved, slice thickness = 3 mm, echo time = 30 ms, repetition time = 2,000 ms, field of view = 240 mm, flip angle = 90°, bandwidth = 2,232 Hz/Px. High-resolution T1 images were obtained in the sagittal direction using a 3D spoiled-gradient-recall sequence (matrix size = 256 × 256, number of slices = 176, slice thickness = 1 mm, echo time = 2.46 ms, repetition time = 1,900 ms, field of view = 250 mm, flip angle = 9°, bandwidth = 170 Hz/Px) after the functional scans.

We pre-processed all fMRI data using Statistical Parametric Mapping 12 (SPM12, http://www.fil.ion.ucl.ac.kr/spm). First, we discarded first five scans for the stabilization of magnetization. Then, we realigned the remaining 750 scans (150 scans a session and five sessions) for each subject via rigid-body transformation without a slice-timing correction. Individual structural images were co-registered to the mean functional image using a rigid-body transformation. Subsequently, functional images were spatially normalized to the Montreal Neurological Institute (MNI) stereotactic standard space and smoothed with a 6-mm full-width at the half-maximum Gaussian kernel. Additionally, we regressed out the nuisance parameters such as six rigid head motion parameters and each mean signal from the white matter and cerebrospinal fluid. Finally, the time series at each voxel were band-pass filtered (0.009–0.08 Hz) to reduce low-frequency drift and physiological noise. The pre-processed data were then used for further statistical analyses.

### Physiological recording and HR

To evaluate physiological responses during the interventions, we acquired physiological data concurrently with fMRI scanning using the Siemens’ built-in equipment. Pulse oximetry data were collected using an MRI-compatible, wireless PPG sensor placed on the right index finger. The sampling rate of the Siemens built-in PPG sensor was 50 Hz, and a time stamp on the output allows temporal registration to the fMRI data. We applied the peak detection algorithm to the PPG time series to identify the beat-to-beat intervals in units of milliseconds. Subsequently, we transformed those beat-to-beat intervals into HR in units of beats per minute. The average HR values were compared using paired sample t-test.

### Seed-based functional connectivity analysis

We calculated FC between the seed regions of interests (ROIs) and the other brain grey matter using a correlation approach. The PCC and ventromedial PFC were selected to investigate default mode rsFC, and we used their coordinates from Dosenbach atlas^57^. The bilateral amygdala and bilateral NA were selected to investigate the networks for emotion and reward-motivation, respectively, and their coordinates are summarized in Supplementary Table S2. For each participant, we computed correlation coefficients between the time series of each ROI and the entire voxels within the grey matter and transformed them to *z*-value using Fisher’s *r*-to-*z* transformation to create connectivity maps. Individual FC for each ROI was computed, and resulting maps were subsequently used in the second-level random effect analysis. First, the FC maps obtained for each ROI were compared between the gratitude intervention and baseline, between the resentment intervention and baseline, and between the gratitude and resentment interventions using paired sample t-test. Second, we conducted repeated-measures ANOVA to explore any significant changes in rsFC among three resting-states: the baseline, after the gratitude intervention, and after the resentment intervention. Significant clusters were determined based on family-wise error (FWE) corrected *P*_FWE_ < 0.05 with a cluster-determining threshold (CDT) at uncorrected *P* < 0.001. For the significant clusters observed in repeated-measures ANOVA, we further conducted *post-hoc* analysis to identify the direction of the differences in all pair-wise comparisons: baseline vs. after-gratitude, baseline vs. after-resentment, and after-gratitude vs. after-resentment. Significant differences were obtained at a threshold of Bonferroni-corrected *P* < 0.05.

### Group independent component analysis and inter-network functional connectivity

Temporal Concatenation Group ICA (TC-GICA) was conducted within the whole brain areas. The procedures for TC-GICA were composed of three steps as described in the previous study^58^. We reduced the pre-processed fMRI data using a two-level principal component analysis. First, the 750 scans for each participant were reduced to 30 principal components (PCs). The 30 PCs at the first level were explained 75 ± 3% of the variance of the five sessions of fMRI data in each subject. Second, a total of 960 temporal components (30 components/subject × 32 subjects) were temporally concatenated to 20 PCs and then unmixed with TC-GICA using infomax algorithm^59^. In agreement with prior studies, the number of components to a lower order TC-GICA was fixed to 20 components^58^. Lastly, spatial independent component (IC) maps and the corresponding time-courses for each subject were extracted using the dual regression approach. Template-matching method was applied to identify IC maps relevant to the current study. Given our hypothesis, we selected five spatial IC maps and the corresponding time-courses matched for the default mode, temporolimbic, salience, and bilateral frontoparietal networks (see Supplementary Figure S5).

### Temporal synchronization between FC and HR during the interventions

To identify temporal synchronization between FC and HR during the interventions, whole-brain sliding-window seed-based FC analysis was performed in each individual space, using 60 s windows and sliding in steps of 10 s, leading to 25 windows across each fMRI scan (Fig. 5B–E). Moreover, we computed sliding-window inter-network FC by applying the same methodology on IC time-courses. Subsequently, we estimated FC-HR synchronization by computing the two-tailed Pearson’s correlation coefficients between sliding-windows FC and sliding-windows fluctuations in HR, yielding a strength of temporal synchronization between FC and HR. Finally, we conducted one-sample t-test to identify significant co-fluctuating patterns of seed-based FC with HR. Significant clusters were determined based on *P*_FWE_ < 0.05 with a primary CDT at uncorrected *P* < 0.005. Also, we computed significant temporal co-fluctuation patterns between sliding-window inter-network FC and HR using one-sample t-test. After correcting multiple comparisons using false discovery rate (FDR), the statistically meaningful results (*P*_FDR_ < 0.1) were obtained, considering that values of *P*_FDR_ in the range of 0.1–0.2 are meaningful in neuroimaging analysis^60^, and we performed *post-hoc* analysis for these results.

### Relationships with emotion and motivation scales

For the NA- and amygdala-based FC maps, we performed linear regression analysis to explore the brain regions that are significantly associated with behavioral variables. Given our hypothesis regarding the selection of the seed regions, the subscale scores of SDT were used to identify relationships between individual’s motivation and NA-based rsFC, whereas the subscale scores of the HADS were used to identify the relationships between the individual’s ability to regulate emotions and amygdala-based rsFC via linear regression analysis. Significant brain regions were determined based on *P*_FWE_ < 0.05 with a primary CDT at uncorrected *P* < 0.005. Furthermore, significant linear relationships between inter-network FC and behavioral variables such as the SDT and HADS scores were identified by two-tailed Pearson’s correlation analysis.

## Electronic supplementary material

Supplementary Information
