# Supplementary material for: Effects of gratitude meditation on neural network functional connectivity and brain-heart coupling
Source: Sci Rep. 2017 Jul 11;7:5058. doi: 10.1038/s41598-017-05520-9 (PMC5506019; doi:10.1038/s41598-017-05520-9)
Supplement: Supplementary file 1 — Supplementary Information [file 41598_2017_5520_MOESM1_ESM.pdf]

Effects of gratitude meditation on neural network functional connectivity and brain-heart coupling

Sunghyon Kyeong, Joohan Kim, Dae-Jin Kim, Hesun Erin Kim, and Jae-Jin Kim

(Supplementary Information)

Supplementary Material S1: Intervention scripts

Followings are English translated script for the gratitude and resentment interventions.

**Supplementary Box 1**. English translated script for the gratitude intervention (originally in Korean)
We will begin the breathing exercise. First, relieve all the tension in your body, and breathe in through your nose, then slowly breathe out through your mouth. Breathe in slowly as you count to three, and do the same when you breathe out. Breathe at your own pace, but make sure to inhale through your nose and exhale through your mouth slowly. From now on, follow the directions while you focus on your breathing as well, however, you may breathe at your own pace. By focusing on our breath, we can train to strengthen our minds and improve our abilities. Concentrate on the air coming in while you are inhaling, and relieve all the tension in your body every time you exhale. When exhaling through your mouth, relax your jaw so that there is a space between your upper teeth and lower teeth. Also, relax your neck and shoulders. Relax your left and right arms, even your wrists as well. As you slowly exhale, relieve all the tension on your back and chest, and feel your body without any tension. Now, you will be thinking about your mother as you breathe deeply. We will be thinking about the mother's love and the gratitude you have for that love. Think of your mother who loved and cared for you since childhood. Maintain your deep breaths while sincerely giving gratitude to your mother. Vividly recall and give thanks all the thankful moments when your mother sacrificed herself for you. Breathe slowly as you take the time to think each one of many nice things your mother has done for you. Be as specific as possible. Try saying a heartfelt gratitude to your mother in your mind. Feel the love and gratitude for our mom and repeatedly say "thank you" in your mind. Thank you for your participation.

**Supplementary Box 2**. English translated script for the resentment intervention (originally in Korean)
We will begin the breathing exercise. First, relieve all the tension in your body, and breathe in through your nose, then slowly breathe out through your mouth. Breathe in slowly as you count to three, and do the same when you breathe out. Breathe at your own pace, but make sure to inhale through your nose and exhale through your mouth slowly. From now on, follow the directions while you focus on your breathing as well, however, you may breathe at your own pace. As you are breathing slowly, remember the time you were angriest and most irritated. Picture the moments that annoy you, even just by thinking about them. Look back at the mistakes you've made in the past. Imagine the faces of the people who anger and bully you. Think about the moments of failure that agonize you. A lot of your wrongdoings and mistakes, in fact, happen because of your own flaws. Thoroughly reflect on the areas that you lack, the things that you are particularly bad at, your weaknesses and vulnerabilities. Think about and reflect on the image of yourself trying to hide your shameful side from others. Think about the shameful side of you that has been hidden deep inside of you. Many times, your mistakes and failures are someone else's fault. Picture the people who usually hate, envy, or bully you. Among these people, think of the one person, in particular, who you resent or loathe the most, in detail. Then, think of all the flaws that person has. Think through how cheap, hypocritical, and how evil the person is. Also think about how cunning, wicked, and selfish the person is. Say this in your mind: "I hate you." Feel the anger and the hatred boiling from deep inside you. Yell "I loathe and despise people like you," in your mind, repeatedly. Say, "I really hate you." As you continue to breathe slowly, pay attention to the anger you are feeling. Thank you for your participation.

Supplementary Material S2: Time intervals between all successive fMRI scans

Supplementary Figure S1 shows the experimental procedure with time stamps to illustrate the time intervals between consecutive fMRI scans, which were present because of differences in the duration of audio instructions before each fMRI scanning. We delivered a simple instruction before starting the intervention fMRI scans and resting-state scans via the built-in microphone. The time interval between the start time of the first intervention fMRI scan (t_2) and end time of the baseline fMRI scan (t_1) can be computed by a subtraction between two time stamps (t_2-t_1). Similarly, we computed the time intervals between all successive fMRI scans.

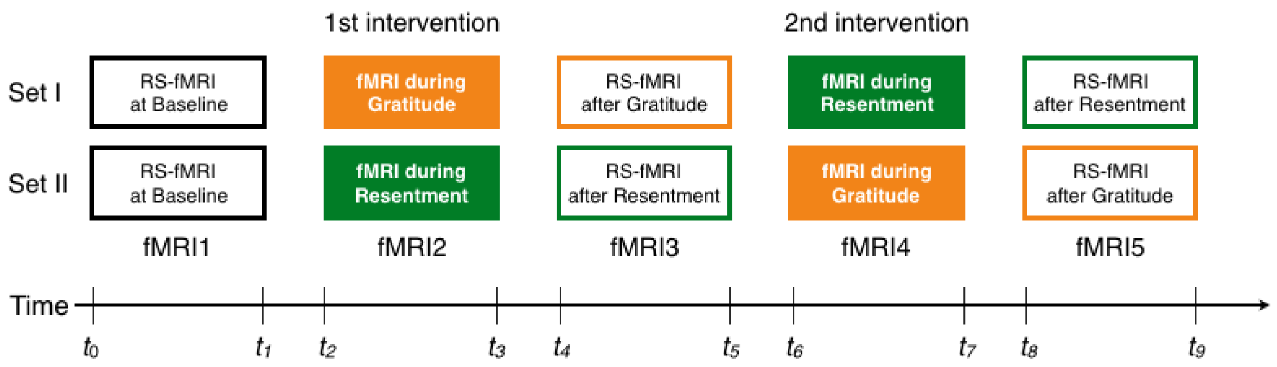

Supplementary Figure S1. Experimental procedure. The order of experiments (set I and II) were counter balanced across participants. Abbreviations: RS-fMRI, resting-state functional magnetic resonance imaging.

The intermissions before fMRI scans were not significantly different between the first intervention (t_2-t_1) and the second intervention (t_6-t_5) and between follow-up resting-states (t_4-t_3 vs. t_8-t_7), but significantly different between the intervention and follow-up resting-state (t_2-t_1 vs. t_4-t_3 or t_6-t_5 vs. t_8-t_7) (Supplementary Figure S2). Meanwhile, the average time intervals between during- and after-intervention fMRI acquisition were 33.0 ± 10.1 and 29.8 ± 10.9 seconds for the gratitude and resentment interventions, respectively, and those intervals were not significantly different between the two interventions (t_{31} = 1.71, p = 0.1).

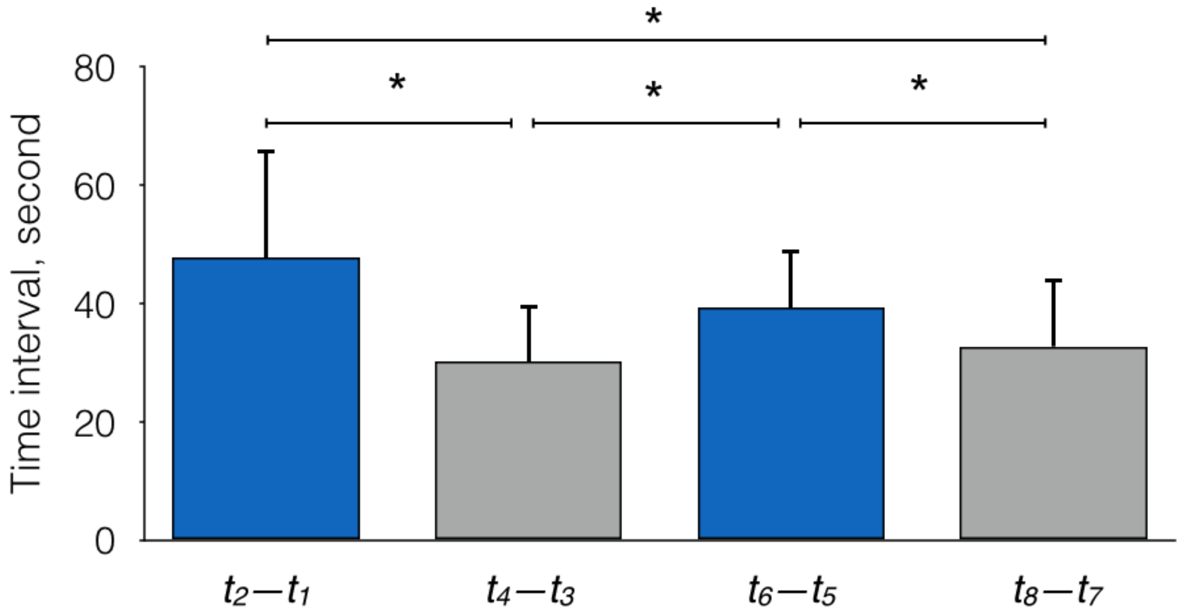

Supplementary Figure S2. The average time intervals for all successive fMRI scans across the participants. Paired sample t-test revealed significant differences among time intervals based on Bonferroni-corrected $P < 0.05$. We indicated the significant differences as asterisks (*). Please refer to Supplementary Figure S1 for the information about the time stamp, t_i. Blue bars indicate the time intervals between the resting-state fMRI scan and intervention fMRI scans, and gray bars indicate time intervals between the intervention fMRI and follow-up resting state fMRI scans.

Supplementary Table S1 shows the summary of the time intervals between all successive fMRI scans. Two-sample t-test revealed that there were no significant differences in the time intervals for the successive fMRI scans between set I and II.

Supplementary Table S1. Mean and standard deviation (SD) of the time intervals between all successive fMRI scans. Information about a time stamp, t_i, and two experimental sets is provided in Supplementary Figure S1

Time intervals	Overall	Set I	Set II	*T*-value	*P*-value
	Mean ± SD	Mean ± SD	Mean ± SD		
between fMRI1 and fMRI2 (t_2-t_1)	47.7 ± 18.2	52.6 ± 22.3	42.2 ± 10.0	1.7	0.106
between fMRI2 and fMRI3 (t_4-t_3)	30.1 ± 9.7	30.8 ± 9.1	29.3 ± 10.5	0.4	0.674
between fMRI3 and fMRI4 (t_6-t_5)	39.3 ± 9.7	39.1 ± 10.5	39.5 ± 9.2	-0.1	0.916
between fMRI4 and fMRI5 (t_8-t_7)	32.7 ± 11.4	30.3 ± 11.6	35.4 ± 10.9	-1.3	0.207

Heart rate data at the baseline, during the interventions, and after the interventions were presented in Supplementary Figure S3. Although we acquired heart rate data for the entire experimental period, we did not compare these heart rate values with heart rate acquired at the baseline because of the following two reasons. First, baseline heart rates were unstable because they were measured immediately after the body-posture change of the participant from stand

up to supine[1]. Second, it may take some time for an individual to stabilize levels of anxiety about closed space (such as MRI scanner) and levels of heart rate until the first (baseline) scanning because heart rate is sensitive to anxiety or emotional stress[2]. Considering these viewpoints, it was a limitation of the current study that participants performed the baseline MRI scanning before they were sufficiently stabilized in the supine position inside the MRI scanner. Therefore, including heart rate at the baseline in the statistical analysis can make it difficult to interpret the results.

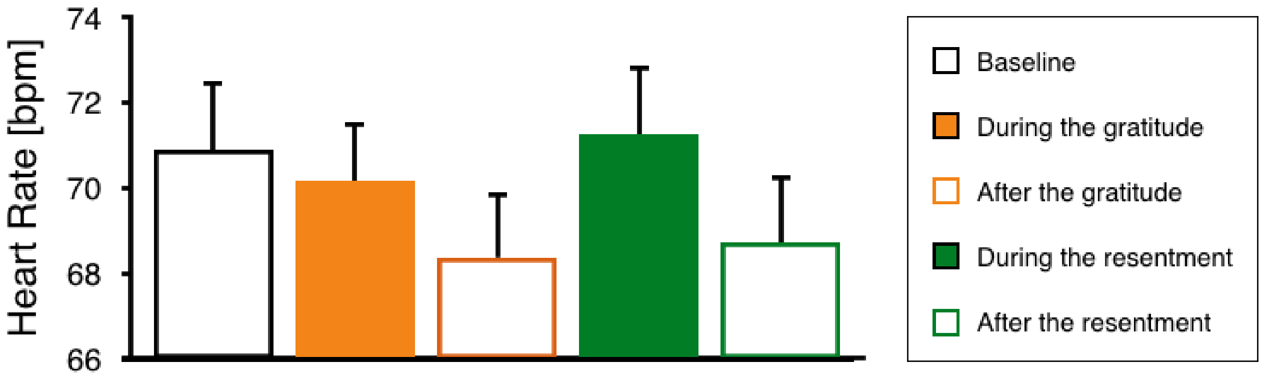

Supplementary Figure S3. Mean and standard error of heart rate at the baseline, during the interventions, and after the interventions.

Meanwhile, we computed heart rate during gratitude session as the 1st intervention and during resentment session as the 1st intervention (the second sessions of Set I and Set II), which were is 68.3 ± 6.9 and 73.4 ± 8.6. Although these two mean values were not statistically different between two sessions (t = -1.76, df=27, p = 0.089), the mean heart rate during gratitude after the baseline is quite lower than mean heart rate during resentment after the baseline (mean difference in heart rate = -5.1 bpm, 95% confidence interval = [-11.0, 0.5] bpm).

Supplementary Material S3: Summary for six seed regions of interests and functional connectivity.

Six seed regions of interests for investigating the default mode, emotion-regulation, and reward-motivation networks are summarized in Supplementary Table S2.

Supplementary Table S2. Information for six seed regions of interests

Region	MNI coordinate			Radius	Reference
	x	y	z		
Posterior cingulate cortex	1	-26	31	6	Dosenbach et al. (2010)[3]
Ventromedial prefrontal cortex	9	51	16	6	Dosenbach et al. (2010)[3]
Lt. Amygdala	-17	-3	-14	6	Goldin et al. (2008)[4]
Rt. Amygdala	19	-5	-14	6	Goldin et al. (2008)[4]
Lt. Nucleus accumbens	-12	8	-8	3	Gu et al. (2010)[5]
Rt. Nucleus accumbens	12	8	-8	3	Gu et al. (2010)[5]

Abbreviation: Lt, left; Rt, right; MNI, Montreal Neurological Institute.

In this section, we provide the detailed summary statistics that were presented in the form of visual illustration in the main manuscript. The supplementary results include temporal synchronization between dynamic functional connectivity and heart rate during the interventions (Supplementary Table S3), changes in seed-based functional connectivity during gratitude and resentment interventions relative to the baseline (Supplementary Tables S4 and S5), and comparisons of resting-state functional connectivity among the baseline, after-gratitude, and after-resentment (Supplementary Table S6). In addition, Supplementary Figure S4 represent the statistical parametric mapping of significant seed-based resting-state functional connectivity for each seed region.

Supplementary Table S3. Temporal synchronization between dynamic functional connectivity and heart rate during the interventions.

Functional connectivity		MNI coordinate			Nvox	Zmax
Seed	Target region	x	y	z		
During Gratitude intervention						
Posterior cingulate cortex	no significant results					
Ventromedial prefrontal cortex	Rt. Paracentral lobule	18	-44	78	205	4.74
	Rt. Angular gyrus	58	-54	40	308	-4.10
	Lt. Lingual gyrus	-6	-84	-10	322	-3.99
Lt. Amygdala	Rt. Superior temporal pole	44	8	-38	261	4.22
	Lt. Superior colliculus	-4	-28	-6	246	4.28

	Rt. Superior occipital gyrus	18	-86	22	164	3.67
	Rt. Cerebellum	8	-52	-8	331	4.57
Rt. Amygdala	no significant results					
Lt. Nucleus accumbens	Lt. Supplementary motor area	-12	-2	66	296	4.03
	Lt. Superior temporal gyrus	-36	-32	24	382	4.80
	Rt. Superior temporal gyrus	44	-38	20	177	4.46
	Rt. Insula	40	10	10	515	4.35
	Lt. Putamen	-34	20	12	705	4.03
	Rt. Supramarginal gyrus	52	-22	26	183	3.91
	Rt. Inferior temporal gyrus	40	-10	-36	170	3.49
Rt. Nucleus accumbens	no significant results					
During Resentment intervention						
Posterior cingulate cortex	no significant results					
Ventromedial prefrontal cortex	no significant results					
Lt. Amygdala	no significant results					
Rt. Amygdala	no significant results					
Lt. Nucleus accumbens	no significant results					
Rt. Nucleus accumbens	no significant results					

Significant clusters were obtained at family-wise error rate corrected $P < 0.05$

Abbreviation: Lt, left; Rt, right; MNI, Montreal Neurological Institute; Nvox, number of contiguous voxels; Zmax, maximum z-value within the cluster.

Supplementary Table S4. Statistical comparisons of seed-based functional connectivity between the gratitude intervention and baseline.

Functional connectivity		MNI coordinate, mm			Nvox	Zmax
Seed	Target region	x	y	z		
Contrast of [during Gratitude > baseline]						
Posterior cingulate cortex	Rt. Dorsolateral prefrontal cortex	44	14	38	319	5.08
	Rt. Caudate	12	-12	16	94	4.44
	Lt. Superior occipital gyrus	-26	-92	34	118	5.10
	Rt. Superior occipital gyrus	48	-74	14	756	5.32
Ventromedial prefrontal cortex	Lt. Dorsolateral prefrontal cortex	-38	42	18	481	6.09
	Lt. Dorsomedial prefrontal cortex	-6	32	46	464	6.16
	Lt. Inferior frontal gyrus	-36	16	0	1507	8.26
	Rt. Inferior frontal gyrus	34	18	6	2664	7.11
	Lt. Precentral gyrus	-36	-8	44	328	6.98
	Lt. Supramarginal gyrus	-60	-26	44	1283	6.09
	Rt. Supramarginal gyrus	38	-48	52	775	7.32
	Rt. Inferior temporal gyrus	58	-50	-4	184	5.33
	Lt. Superior occipital gyrus	-38	-78	20	216	5.30
Lt. Amygdala	not significant					
Rt. Amygdala	not significant					
Lt. Nucleus accumbens	Rt. Middle temporal gyrus	40	-82	20	91	5.72
Rt. Nucleus accumbens	Rt. Angular gyrus	64	-60	12	217	5.27

Contrast of [during Gratitude < baseline]						
Posterior cingulate cortex	Lt. Cuneus	-12	-96	-8	214	5.55
	Rt. Cuneus	16	-98	-2	224	5.34
Ventromedial prefrontal cortex	Rt. Orbitofrontal cortex	2	60	-8	183	4.70
	Lt. Middle temporal gyrus	-48	-34	-2	952	6.60
	Rt. Inferior temporal pole	50	10	-26	239	5.52
	Rt. Middle temporal gyrus	46	-20	-6	115	4.78
	Rt. Middle temporal gyrus	58	-8	-8	106	4.69
	Lt. Angular gyrus	-62	-52	24	270	4.83
Lt. Amygdala	not significant					
Rt. Amygdala	not significant					
Lt. Nucleus accumbens	not significant					
Rt. Nucleus accumbens	Lt. Fusiform gyrus	-16	-88	-6	1623	7.10
	Rt. Fusiform gyrus	28	-72	-10	1005	6.68

Significant clusters were obtained at family-wise error rate corrected $P < 0.05$.
Abbreviation: Lt, left; MNI, Montreal Neurological Institute; Nvox, number of contiguous voxels; Rt, right; Zmax, maximum z-value within the cluster.

Supplementary Table S5. Statistical comparisons of seed-based functional connectivity between the resentment intervention and baseline.

Functional connectivity		MNI coordinate, mm			Nvox	Zmax
Seed	Target region	x	y	z		
Contrast of [during Resentment > baseline]						
Posterior cingulate cortex	Rt. Dorsolateral prefrontal cortex	54	42	18	217	5.83
	Rt. Ventrolateral prefrontal cortex	38	14	26	283	5.05
	Rt. Insula	40	18	0	1031	5.72
	Lt. Superior parietal lobule	-30	-60	64	322	5.28
	Lt. Supramarginal gyrus	-66	-34	26	251	4.65
	Rt. Supramarginal gyrus	68	-24	26	367	5.04
	Lt. Middle occipital gyrus	-30	-92	22	452	5.96
	Rt. Middle temporal gyrus	44	-70	10	154	4.89
	Lt. Putamen	-36	-8	-8	343	5.88
	Lt. Thalamus	-14	-10	10	156	5.16
	Rt. Thalamus	12	-18	8	124	4.86
Ventromedial prefrontal cortex	Rt. Dorsolateral prefrontal cortex	46	36	22	1166	6.71
	Lt. Inferior frontal gyrus	-32	18	8	4283	12.25
	Rt. Inferior frontal gyrus	34	22	4	5341	9.82
	Rt. Postcentral gyrus	58	-18	34	129	5.02
	Lt. Supramarginal gyrus	-26	-46	44	1647	6.01
	Rt. Supramarginal gyrus	56	-38	46	872	4.96
	Lt. Middle occipital gyrus	-32	-72	26	226	5.73
	Lt. Cerebellum	-6	-70	-24	177	6.30
	Rt. Cerebellum	30	-68	-16	115	6.44
Lt. Amygdala	not significant					
Rt. Amygdala	not significant					
Lt. Nucleus accumbens	not significant					
Rt. Nucleus accumbens	Lt. Precuneus	0	-40	48	112	5.12
Contrast of [during Resentment < baseline]						
Posterior cingulate cortex	Rt. Ventromedial prefrontal cortex	6	56	-2	396	5.82
	Lt. Precuneus / posterior cingulate cortex	-12	-54	26	2304	9.64

	Lt. Angular gyrus	-38	-64	32	556	6.57
	Rt. Angular gyrus	48	-60	26	658	5.69
	Lt. Middle temporal gyrus	-56	-4	-24	254	6.17
Ventromedial prefrontal cortex	Rt. Middle temporal pole	50	12	-28	659	8.69
	Rt. Ventromedial prefrontal cortex	4	52	-14	956	8.21
	Lt. Angular gyrus	-42	-56	26	899	7.66
	Rt. Angular gyrus	50	-58	26	955	7.47
	Rt. Middle temporal pole	56	-34	0	593	6.99
	Lt. Middle temporal gyrus	-56	6	-22	1844	6.83
	Rt. Precuneus / posterior cingulate cortex	8	-58	38	2283	6.77
	Lt. Middle occipital gyrus	-38	-82	40	103	4.45
Lt. Amygdala	Lt. Cuneus	-10	-108	4	159	5.42
Rt. Amygdala	not significant					
Lt. Nucleus accumbens	not significant					
Rt. Nucleus accumbens	Lt. Fusiform gyrus	-14	-88	-8	831	5.43
	Rt. Fusiform gyrus	20	-90	-8	647	6.40

Significant clusters were obtained at family-wise error rate corrected $P < 0.05$.
Abbreviation: Lt, left; MNI, Montreal Neurological Institute; Nvox, number of contiguous voxels; Rt, right; Zmax, maximum z-value within the cluster.

Supplementary Table S6. Statistical comparisons of functional connectivity among resting-states at the baseline, after the gratitude intervention, and after the resentment intervention.

Functional connectivity		MNI coordinate			Baseline, (B)	after Gratitude (G)	after Resentment (R)	RM-ANOVA		Post-hoc analysis†
Seed	Target region	x	y	z	Mean ± SD	Mean ± SD	Mean ± SD	Nvox	F	
PCC	Rt. DMPFC	2	20	48	-0.03 ± 0.17	0.16 ± 0.18	0.09 ± 0.20	315	20.1	G > B, R > B
	Rt. Orbitofrontal cortex	6	46	-10	0.11 ± 0.16	-0.06 ± 0.17	-0.02 ± 0.15	453	24.4	G < B, R < B
	Rt. DLPFC	28	56	28	-0.03 ± 0.15	0.12 ± 0.17	0.09 ± 0.15	176	13.1	G > B, R > B
	Lt. Precuneus	-4	-62	24	0.30 ± 0.20	0.08 ± 0.21	0.17 ± 0.18	1884	35.5	G<B, R<B, G<R
	Lt. Angular gyrus	-44	-56	22	0.16 ± 0.19	-0.08 ± 0.16	-0.04 ± 0.22	1055	26.2	G > B, R > B
	Lt. Supramarginal gyrus	-56	-44	52	-0.03 ± 0.16	0.12 ± 0.15	0.11 ± 0.18	160	15.7	G > B, R > B
	Rt. Angular gyrus	40	-58	28	0.17 ± 0.17	0.04 ± 0.21	0.02 ± 0.20	444	16.7	G > B, R > B
	Rt. Cuneus	4	-88	4	-0.05 ± 0.16	0.09 ± 0.14	-0.05 ± 0.15	83	12.1	G > B, G > R
	Lt. Middle temporal gyrus	-54	-8	-16	0.12 ± 0.15	-0.09 ± 0.16	-0.03 ± 0.17	717	29.4	G < B, R < B
	Rt. Middle temporal gyrus	56	2	-22	0.11 ± 0.17	-0.10 ± 0.17	-0.02 ± 0.17	311	19.9	G > B, R > B
	Lt. Superior temporal gyrus	-68	-34	-4	0.11 ± 0.13	-0.03 ± 0.14	0.06 ± 0.11	182	17.9	G < B, G < R
	Rt. Putamen	20	6	6	-0.08 ± 0.17	0.12 ± 0.19	0.10 ± 0.17	2192	25.2	G > B, R > B
VMPFC	Rt. DLPFC	42	42	16	-0.30 ± 0.25	-0.17 ± 0.24	-0.14 ± 0.25	107	16.1	G > B, R > B
	Lt. Supramarginal gyrus	-58	-26	44	-0.36 ± 0.24	-0.21 ± 0.14	-0.20 ± 0.18	200	14.2	G > B, R > B
	Rt. Angular gyrus	48	-56	24	0.46 ± 0.20	0.40 ± 0.23	0.30 ± 0.26	158	15.0	R < B, G > R
	Lt. Precuneus	-20	-52	50	-0.27 ± 0.16	-0.10 ± 0.21	-0.12 ± 0.21	142	13.4	G > B, R > B
	Lt. Cuneus	-10	-90	4	-0.16 ± 0.14	0.01 ± 0.18	-0.01 ± 0.22	123	14.9	G > B, R > B
	Rt. Fusiform gyrus	32	-66	-16	-0.17 ± 0.16	-0.13 ± 0.17	-0.03 ± 0.20	110	13.6	R > B, G < R
	Lt. Lateral occipital	-34	-76	16	-0.24 ± 0.15	-0.14 ± 0.21	-0.08 ± 0.21	136	13.8	G > B, R > B
	Rt. Lateral occipital	36	-76	16	-0.20 ± 0.17	-0.11 ± 0.20	-0.06 ± 0.19	128	11.9	G > B, R > B
	Lt. Middle temporal gyrus	-52	-18	-16	0.34 ± 0.18	0.20 ± 0.20	0.20 ± 0.23	290	18.2	G < B, R < B
	Lt. Cerebellum	-10	-68	-20	-0.19 ± 0.14	-0.03 ± 0.16	-0.07 ± 0.16	148	14.7	G > B, R > B
	Rt. Cerebellum	4	-68	-22	-0.12 ± 0.13	0.02 ± 0.17	-0.08 ± 0.18	74	23.1	G > B, G > R
Lt. AMY	no significant results									
Rt. AMY	Lt. VLPFC	-56	-2	8	-0.02 ± 0.17	0.12 ± 0.18	0.04 ± 0.13	75	18.5	G > B
Lt. NA	Rt. Precuneus	6	-78	38	-0.06 ± 0.13	0.05 ± 0.14	0.08 ± 0.16	97	27.7	G > B, R > B

Rt. NA	Lt. VLPFC	-30	36	12	-0.07 ± 0.09	0.02 ± 0.15	-0.09 ± 0.14	79	18.4	G > B, G > R
	Rt. VLPFC	38	44	2	-0.13 ± 0.14	-0.07 ± 0.18	-0.20 ± 0.16	92	13.1	G > R
	Rt. Middle temporal gyrus	54	-62	2	0.07 ± 0.15	-0.08 ± 0.17	0.05 ± 0.20	208	15.1	G < B, G < R
	Lt. Superior temporal gyrus	-38	-36	12	0.03 ± 0.11	-0.07 ± 0.16	0.06 ± 0.17	67	14.7	G < B, G < R
	Rt. Superior temporal gyrus	64	-24	14	-0.01 ± 0.15	-0.13 ± 0.16	0.04 ± 0.16	92	12.9	G < B, G < R

Abbreviations: Lt, left; Rt, right; PCC, posterior cingulate cortex; VMPFC, ventromedial prefrontal cortex; AMY, amygdala; NA, nucleus accumbens; DMPFC, dorsomedial prefrontal cortex; DLPFC, dorsolateral prefrontal cortex; VLPFC, ventrolateral prefrontal cortex; MNI, Montreal Neurological Institute; Nvox, number of contiguous voxels; Zmax, maximum z-value within the cluster; RM-ANOVA, repeated-measure analysis of variance; Nvox, number of voxel within a cluster; SD, standard deviation.

† A significance level of post-hoc analysis among three sessions was corrected for multiple-comparisons using Bonferroni method.

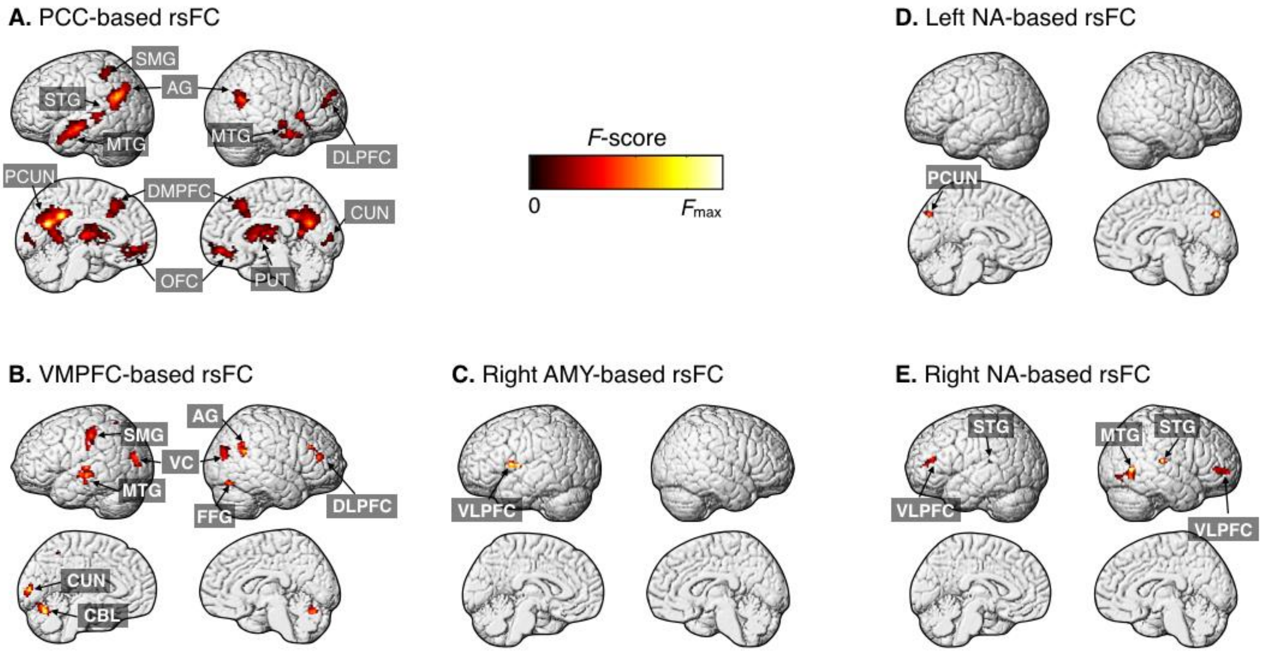

Supplementary Figure S4. Significant seed-based resting-state functional connectivity (rsFC) when the seed was (A) the posterior cingulate cortex (PCC), (B) ventromedial prefrontal cortex (VMPFC), (C) right amygdala (AMY), (D) left nucleus accumbens (NA), and (E) right NA. Abbreviations: AG, angular gyrus; CBL, cerebellum; CUN, cuneus; DLPFC, dorsolateral prefrontal cortex; DMPFC, dorsomedial prefrontal cortex; FFG, fusiform gyrus; MTG, middle temporal gyrus; OFC, orbitofrontal gyrus; PCUN, precuneus; PUT, putamen; SMG, supramarginal gyrus; STG, superior temporal gyrus; VC, visual cortex; VLPFC, ventrolateral prefrontal cortex.

Supplementary Material S4: Inter-network functional connectivity

In order to investigate inter-network functional connectivity, we produced spatial independent component maps and the corresponding time-courses using independent component analysis and spatiotemporal dual-regression. Ten independent component maps (Supplementary Figure S5) were identified using the template-matching method and were matched with the previously reported resting-state networks[654433](Smith et al., 2009)(Smith et al., 2009).[6] Among them, given our hypothesis, five networks colored in cyan such as the default mode, auditory, salience, and bilateral frontoparietal networks, were selected for the effects of gratitude and resentment interventions on inter-network functional connectivity. In this section, inter-network functional connectivity was evaluated using the time-courses obtained from the dual-regression analysis.

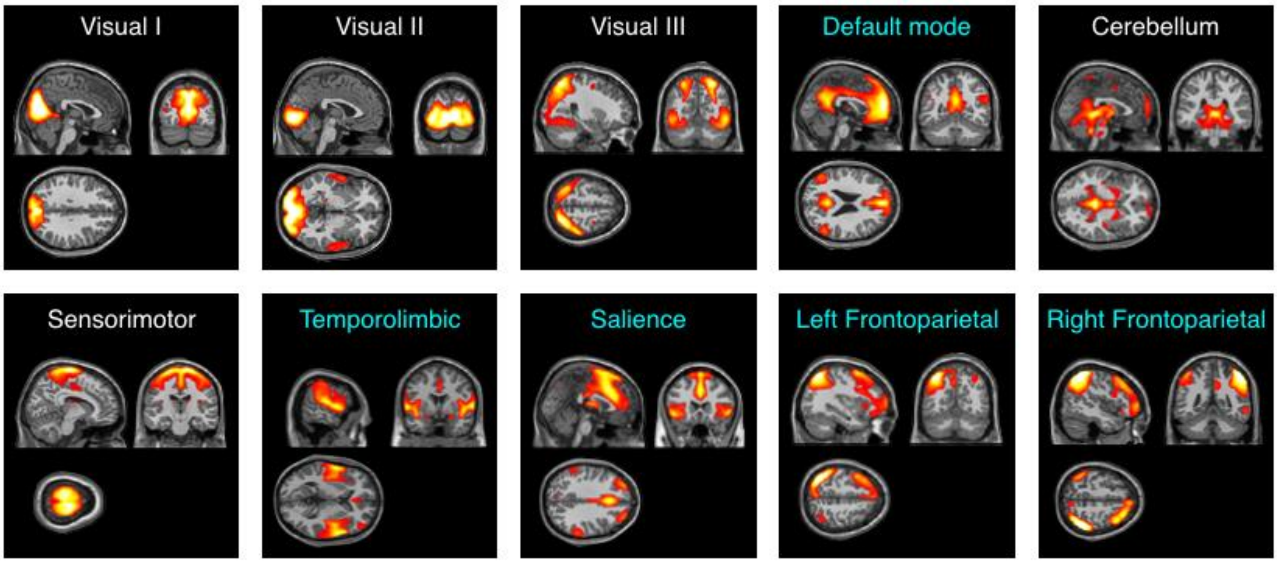

Supplementary Figure S5. Ten independent component maps for the resting-state networks.

Subsequently, temporal synchronization between dynamic inter-network functional connectivity and heart rate were counted during the gratitude and resentment interventions (Supplementary Table S7). Inter-network functional connectivity was compared between the interventions and baseline (Supplementary Table S8), between the gratitude and resentment interventions (Supplementary Table S9), and between resting-states at the baseline, after the gratitude intervention, and after the resentment intervention (Supplementary Table S10).

Supplementary Table S7. Temporal synchronization between dynamic inter-network functional connectivity and heart rate during the interventions.

Inter-network functional connectivity	during Gratitude			during Resentment		
	Mean ± SD	*T*	P_{FDR}	Mean ± SD	*T*	P_{FDR}
Default mode – Temporolimbic network	-0.14 ± 0.528	-1.4	0.31	0.08 ± 0.532	0.9	0.60
Default mode – Salience network	-0.10 ± 0.533	-1.1	0.37	0.31 ± 0.501	3.3	0.03
Default mode – Left frontoparietal	-0.13 ± 0.516	-1.4	0.31	0.02 0.486	0.3	0.88
Default mode – Right frontoparietal	0.09 ± 0.530	0.9	0.41	0.17 ± 0.437	2.0	0.26
Temporolimbic network – Salience network	-0.11 ± 0.507	-1.2	0.34	-0.09 ± 0.514	-1.0	0.60
Temporolimbic network – Left frontoparietal	-0.19 ± 0.465	-2.2	0.16	-0.08 ± 0.516	-0.8	0.60
Temporolimbic network – Right frontoparietal	-0.22 ± 0.561	-2.1	0.16	-0.01 ± 0.423	-0.1	0.90
Salience network – Left frontoparietal	-0.20 ± 0.386	-2.8	0.09	0.08 ± 0.442	1.0	0.60
Salience network – Right frontoparietal	-0.12 ± 0.467	-1.4	0.31	0.09 ± 0.466	1.1	0.60
Left frontoparietal – Right frontoparietal	-0.06 ± 0.568	-0.6	0.57	-0.05 ± 0.461	-0.6	0.70

Mean and standard deviation (SD) are provided together with the statistical significance obtained from one-sample t-test. The statistical significances were controlled for multiple comparison using false discovery rate (FDR) corrected P-value (P_{FDR}).

Supplementary Table S8. Comparison between inter-network functional connectivity during the interventions and at the baseline.

Inter-network functional connectivity	Intervention	Baseline	T(df=31)	P_{FDR}
	Mean ± SD	Mean ± SD		
during Gratitude intervention vs. Baseline				
Default mode – Temporolimbic network	-0.01 ± 0.35	-0.43 ± 0.32	6.25	<0.001
Default mode – Salience network	0.19 ± 0.32	-0.17 ± 0.29	6.19	<0.001
Default mode – Left frontoparietal	0.36 ± 0.27	0.18 ± 0.30	2.85	0.010
Default mode – Right frontoparietal	0.41 ± 0.23	0.18 ± 0.26	4.39	<0.001
Temporolimbic network – Salience network	0.57 ± 0.24	0.54 ± 0.24	0.63	0.534
Temporolimbic network – Left frontoparietal	0.24 ± 0.27	-0.07 ± 0.21	6.28	<0.001
Temporolimbic network – Right frontoparietal	0.17 ± 0.27	-0.08 ± 0.25	4.28	<0.001
Salience network – Left frontoparietal	0.42 ± 0.28	0.15 ± 0.26	4.00	<0.001
Salience network – Right frontoparietal	0.43 ± 0.28	0.06 ± 0.23	5.90	<0.001
Left frontoparietal – Right frontoparietal	0.64 ± 0.26	0.55 ± 0.26	1.45	0.176
During Resentment intervention vs. baseline				
Default mode – Temporolimbic network	0.08 ± 0.34	-0.43 ± 0.32	6.77	<0.001
Default mode – Salience network	0.40 ± 0.34	-0.17 ± 0.29	7.70	<0.001
Default mode – Left frontoparietal	0.33 ± 0.33	0.18 ± 0.30	2.16	0.049
Default mode – Right frontoparietal	0.61 ± 0.28	0.18 ± 0.26	7.87	<0.001
Temporolimbic network – Salience network	0.54 ± 0.27	0.54 ± 0.24	0.01	0.993
Temporolimbic network – Left frontoparietal	0.31 ± 0.21	-0.07 ± 0.21	9.40	<0.001
Temporolimbic network – Right frontoparietal	0.21 ± 0.26	-0.08 ± 0.25	4.53	<0.001
Salience network – Left frontoparietal	0.50 ± 0.28	0.15 ± 0.26	5.30	<0.001
Salience network – Right frontoparietal	0.51 ± 0.33	0.06 ± 0.23	6.27	<0.001
Left frontoparietal – Right frontoparietal	0.48 ± 0.31	0.55 ± 0.26	-1.05	0.334

Mean and standard deviation (SD) are provided together with the statistics obtained from paired-sample t-test. The statistical significances were controlled for multiple comparison using false discovery rate (FDR) corrected *P*-value (P_{FDR}). Abbreviation: df, degree of freedom.

Supplementary Table S9. Inter-network functional connectivity during the interventions.

Inter-network functional connectivity	during Gratitude<br>Mean ± SD	during Resentment<br>Mean ± SD	T (df=31)	P_{FDR}
Default mode – Temporolimbic network	-0.01 ± 0.35	0.08 ± 0.34	-1.54	0.222
Default mode – Salience network	0.19 ± 0.32	0.40 ± 0.34	-4.01	0.003
Default mode – Left frontoparietal	0.36 ± 0.27	0.33 ± 0.33	0.63	0.531
Default mode – Right frontoparietal	0.41 ± 0.23	0.61 ± 0.28	-3.79	0.003
Temporolimbic network – Salience network	0.57 ± 0.24	0.54 ± 0.27	0.68	0.531
Temporolimbic network – Left frontoparietal	0.24 ± 0.27	0.31 ± 0.21	-1.73	0.222
Temporolimbic network – Right frontoparietal	0.17 ± 0.27	0.21 ± 0.26	-0.77	0.531
Salience network – Left frontoparietal	0.42 ± 0.28	0.50 ± 0.28	-1.46	0.222
Salience network – Right frontoparietal	0.43 ± 0.28	0.51 ± 0.33	-1.46	0.222
Left frontoparietal – Right frontoparietal	0.64 ± 0.26	0.48 ± 0.31	2.92	0.022

Mean and standard deviation (SD) are provided together with the statistics obtained from paired-sample t-test. The statistical significances were controlled for multiple comparison using false discovery rate (FDR) corrected P-value (P_{FDR}). Abbreviation: df, degree of freedom.

Supplementary Table S10. Inter-network functional connectivity at the baseline and after the interventions.

Inter-network functional connectivity	Baseline	after Gratitude	after Resentment	$F_{2,62}$	P_{FDR}
	Mean ± SD	Mean ± SD	Mean ± SD		
Default mode – Temporolimbic network	-0.43 ± 0.32	-0.15 ± 0.36	-0.17 ± 0.40	10.83	0.001
Default mode – Salience network	-0.17 ± 0.29	0.01 ± 0.27	-0.05 ± 0.31	4.54	0.029
Default mode – Left frontoparietal	0.18 ± 0.30	0.22 ± 0.30	0.20 ± 0.30	0.14	0.868
Default mode – Right frontoparietal	0.18 ± 0.26	0.23 ± 0.28	0.23 ± 0.30	0.85	0.479
Temporolimbic network – Salience network	0.54 ± 0.24	0.44 ± 0.31	0.48 ± 0.24	1.43	0.310
Temporolimbic network – Left frontoparietal	-0.07 ± 0.21	-0.02 ± 0.30	0.08 ± 0.31	4.64	0.029
Temporolimbic network – Right frontoparietal	-0.08 ± 0.25	0.06 ± 0.29	0.13 ± 0.34	7.49	0.006
Salience network – Left frontoparietal	0.14 ± 0.26	0.20 ± 0.29	0.32 ± 0.26	6.12	0.013
Salience network – Right frontoparietal	0.06 ± 0.23	0.14 ± 0.26	0.21 ± 0.27	2.67	0.110
Left frontoparietal – Right frontoparietal	0.55 ± 0.26	0.57 ± 0.27	0.67 ± 0.26	3.29	0.073

Mean and standard deviation (SD) are provided together with the statistics obtained from repeated-measure analysis of variance test. The statistical significances were controlled for multiple comparison using false discovery rate (FDR) corrected P-value (P_{FDR}).

References

1. Bloomfield, D. M. *et al.* Passive head-up tilt and actively standing up produce similar overall changes in autonomic balance. *Am. Heart J.* **134**, 316-320 (1997).

2. Dikecligil, G. N. and Mujica-Parodi, L. R. Ambulatory and challenge-associated heart rate variability measures predict cardiac responses to real-world acute emotional stress. *Biol. Psychiatry* **67**, 1185-1190 (2010).

3. Dosenbach, N. U. *et al.* Prediction of individual brain maturity using fMRI. *Science* **329**, 1358-1361 (2010).

4. Goldin, P. R., McRae, K., Ramel, W. & Gross, J. J. The neural bases of emotion regulation: reappraisal and suppression of negative emotion. *Biol. Psychiatry* **63**, 577-586 (2008).

5. Gu, H. *et al.* Mesocorticolimbic circuits are impaired in chronic cocaine users as demonstrated by resting-state functional connectivity. *NeuroImage* **53**, 593-601 (2010).

6. Smith, S. M. *et al.* Correspondence of the brain's functional architecture during activation and rest. *Proc. Natl. Acad. Sci. U. S. A.* **106**, 13040-13045 (2009).
